# An Automated Summarization Assessment Algorithm for Identifying Summarizing Strategies

**DOI:** 10.1371/journal.pone.0145809

**Published:** 2016-01-06

**Authors:** Asad Abdi, Norisma Idris, Rasim M. Alguliyev, Ramiz M. Aliguliyev

**Affiliations:** 1 Department of Artificial Intelligence Faculty of Computer Science and Information Technology, University of Malaya, 50603, Kuala Lumpur, Malaysia; 2 Institute of Information Technology, Azerbaijan National Academy of Sciences, 9, B. Vahabzade Street, AZ1141 Baku, Azerbaijan; Garvan Institute of Medical Research, AUSTRALIA

## Abstract

**Background:**

Summarization is a process to select important information from a source text. Summarizing strategies are the core cognitive processes in summarization activity. Since summarization can be important as a tool to improve comprehension, it has attracted interest of teachers for teaching summary writing through direct instruction. To do this, they need to review and assess the students' summaries and these tasks are very time-consuming. Thus, a computer-assisted assessment can be used to help teachers to conduct this task more effectively.

**Design/Results:**

This paper aims to propose an algorithm based on the combination of semantic relations between words and their syntactic composition to identify summarizing strategies employed by students in summary writing. An innovative aspect of our algorithm lies in its ability to identify summarizing strategies at the syntactic and semantic levels. The efficiency of the algorithm is measured in terms of Precision, Recall and F-measure. We then implemented the algorithm for the automated summarization assessment system that can be used to identify the summarizing strategies used by students in summary writing.

## Introduction

Reading skills are essential for success in society. Reading affects different aspects in our life, especially in school. The aim of reading is to elicit meaning from the written text. A lack of capacity in this area may affect the comprehension ability. Comprehension involves inferential and evaluative thinking, not just a reproduction of the author's words. It can be taught and improved through teaching students during their learning process.

Recently, the results of some studies have shown that summarization can be an important key for reading comprehension. Summarization is the process of automatically producing a compressed version of a given text that provides useful information for the user [1, 2, 3, 4].

Summarizing strategies are the core of the cognitive processes involved in the summarization activity. These include a set of conscious tasks that are used to create a summary text. There are several summarizing strategies to determine important information, eliminate irrelevant information, and extract the main idea of a source text. According to the result of some studies, a major difficulty faced by students in summary writing is the lack of skills in applying summarizing strategies.

Since summarization can be used as a measure of understanding in school [5, 6], it has attracted interest of teachers for teaching summary writing through Direct Instruction [5]. Where teachers need to possess some information, such as what summarizing strategies are used by students, the ability of students to use summarizing strategies, how students use summarizing strategies, and their students’ weaknesses in summarizing. To collect this information manually is difficult and very time-consuming. On the other hand, in order to reduce the time they should spend on this task; many teachers choose to reduce the number of summary writing exercises given to their students. Thus, students do not have sufficient practice, which may affect their summary writing ability. To tackle these problems, Computer-Assisted Assessment (CAA), using syntactic and semantic contribution relations is proposed.

Due to the rapid advances in computer, educational researchers have developed methods, tools and self-learning tools [7, 8]. In other hand, due to the progress in other areas, such as e-learning, information extraction and Natural Language Processing, the automatic evaluation of summary writing has been made possible.

This paper is not concerned with the summarization process, where the outcome is a summary text, but with the summarization assessment process, where the result is identifying summarizing strategies. Although previous systems have been developed to assess summarization, most of them focus only on the content coverage. A few systems have been developed to identify summarizing strategies used by students. However, these systems are not able to identify summarizing strategies at the syntactic and semantic levels. Thus, we aim to develop an algorithm for the automated summarization assessment system that can be used to identify the strategies employed by students in summary writing. The proposed algorithm is called ISSLK: Identifying Summarizing Strategies based on Linguistic Knowledge.

The algorithm is based on linguistic knowledge, a combination of semantic relations between words and their syntactic composition. An innovative aspect of our algorithm lies in its ability to identify summarizing strategies syntactically and semantically. In addition, it is able to identify the synonym or similar words among all sentences using a lexical database, WordNet. It is very important to consider this aspect (identifying the synonym or similar words) when evaluating the summaries [9, 10]. The objective of our study is to find a reply to the following research questions: 1) How can the summarizing strategies be identified; 2) How can algorithms to detect text relevancy and identify summarizing strategies be formulated; 3) What is the performance of the algorithm when compared to human judgment?

## Summarizing Strategies Identification

This section presents a set of heuristic rules to identify the summarizing strategies in summary writing. Summarization is a learning strategy that can help students construct and retain a short summary of the important information from the source text. Summarizing strategies are the core of the cognitive process in summary writing [11]. They include a set of conscious tasks to recognize what is important and what is not, to extract the main idea of a source text. Hence, it helps the summarizer to generate an appropriate summary. Different researchers use different terminology to describe the summarizing strategies, which are fundamentally a similar process. These authors [11, 12, 13, 14, 15] suggest several summarizing strategies involved in producing appropriate summaries. These strategies are explained in detail as follows:

### Deletion

To produce a summary sentence, a deletion strategy is used to remove unnecessary information in the sentence of the source text. Unnecessary information includes trivial details about the topics such as *examples* and *scenarios* or redundant information containing the rewording of some of the important information.

### Sentence Combination

To produce a summary sentence, sentence combination is used to combine two or more sentences/phrases from the source text. In other words, phrases from more than one sentence are merged into a summary sentence. These sentences are usually combined using conjunction words, such as *for*, *but*, *and*, *after*, *since*, *and before*.

### Generalization

The generalization rule replaces a general term for a list. There are two kinds of replacement. One is the replacement of a general word for a list of similar items, e.g. ‘*pineapple*, *banana*, *star fruit and pear’* can be replaced by ‘*fruits’*. The other one is the replacement of a general word for a list of similar actions, e.g. the sentences: ‘*Yang eats a pear’*, and ‘*Chen eats a banana’*, can be replaced by: ‘*The boys eat fruits’*.

### Paraphrasing

In the paraphrasing process, a word in the source sentence is replaced with a synonymous word (a different word with the same meaning) in the summary sentence.

### Topic Sentence Selection (TSS)

To produce a summary sentence, the topic sentence selection strategy is used to extract an important sentence from the original text to represent the main idea of a paragraph. There are four methods to identify the important sentence:

#### Key method

The most frequent words in a text are the most representative of its content, thus a segment of text containing them is more relevant [16]. Word frequency is a method used to identify keywords that are non-stop-words, which occur frequently in a document [17, 18]. According to [19], sentences having keywords or content words have a greater chance of being included in the summary.

#### Location method

Important sentences are normally at the beginning and the end of a document or paragraphs, as well as immediately below section headings [20, 21]. Paragraphs at the beginning and end of a document are more likely to contain material that is useful for a summary, especially the first and last sentences of the paragraphs [19, 22].

#### Title method

Important sentences normally contain words that are presented in the title and major headings of a document [20]. Thus, words occurring in the title are good candidates for document specific concepts [23].

#### Cue method

Cue phrases are words and phrases that directly signal the structure of a discourse. They are also known as discourse markers, discourse connectives, and discourse particles in computational linguistics [24]. Cue phrases, such as “conclusion” or “in particular” are often followed by important information. Thus, sentences that contain one or more of these cue phrases are considered more important than sentences without cue phrases [25]. These cue words are context dependent. However, due to the existence of different types of text, such as scientific articles and newspaper articles, it is difficult to collect these cue words as a unique list. Hence, since discourse markers can be used as an indicator of important content in a text and are more generic [26], we provide the list using discourse markers. These discourse markers are collected from the previous works [16]. Table 1 shows some of these cue words that may appear in a sentence.

**Table 1:** Sample of cue word list.

Cue words				
As a conclusion	Last of all	Because of that	Hardly	Summarize
As a consequence	As a logical	Result	The paper describe	Consequence
It can be concluded that	Of course	End, therefore	Because of this	Consequently
As a consequence of	As a result	Eventually	Significantly	Thereby
Because our investigation	On that condition	Thus	Conclusion	For this reason

### Invention

A summary sentence is created using invention rule if one makes explicit topic sentences by using his or her own words to state the implicit main idea of the paragraphs. Thus, the invention rule requires that students “add information rather than just delete, select or manipulate sentences already provided for them” [13, 15].

### Copy–verbatim

In the copy-verbatim process, a summary sentence is produced from the source sentence without any changes. This strategy is not part of the summarizing strategies but it is used by students.

In this work, we consider five basic summarizing strategies–sentence combination, deletion, paraphrase, copy–verbatim, topic sentence selection–and four methods–key method, title method, cue method and location method. Since summarizing strategies are general rules and quite ambiguous for the computer to process; hence, we need to transform these general rules into a set of comprehensible rules for processing. For example, an explanation of deletion strategy is as follows:

$$\begin{array}{l} \mathrm{Rule}\;\mathrm{Process}\\ Deletion\;\text{remove unnecessary information}\;from\;the\;original\;text. \end{array}$$

The term *“unnecessary information*” in the example above is very subjective and quite ambiguous for the computer to process and execute. To develop a system that can identify summarizing strategies in summary writing automatically, we need to produce more measurable and precise rules for each summarizing strategy. For this purpose, an analysis has been done on human–written summary. The results of the analysis are used to formulate a more detail and precise rules on how to identify each strategy. In this study, we used the same dataset as described in section “*Experimental evaluations”*. Two experts: a) An English teacher with good reading skills and understanding ability in the English language as well as experience in teaching summary writing; b) A lecturer with experience in using the skills in their teaching method, were asked to identify the summarizing strategies used by summarizer in each summary sentence. The human expert disassembled the summary text into a number of sentences, and then compared each sentence of summary text with all sentences from the original text to determine whether two sentences are semantically identical or not. Semantically identical sentences include same information or talk about similar idea. However, the sentence(s) from the original text that is/are semantically equivalent with the current sentence of summary text can be considered as the source sentence(s) that has/have been associated to produce the current summary sentence. Given two sentences, the summary sentence and the source sentence, the experts determine the summarizing strategies employed by summarizer to produce the current sentence of summary text.

Table 2 displays an overview of the analysis that we have conducted on summary text. It illustrates the results achieved over the summaries. In particular, for each summary text, the number of each sentence of summary text is shown in the first column; while the second column presents the summary sentences, the third column displays the most relevant sentences which are extracted from the source text and have been used to produce summary sentences; and finally the last column shows the summarizing strategies that have been employed to produce each summary sentence. This study aims to determine most relevant sentences from the original text for each summary sentence and identify the summarizing strategies used to construct the summary sentence.

**Table 2:** Analysis on summary sentences.

No. of sentence	Summary sentence	Original sentence	Summarizing strategy
1	“The currents kept pushing the boat further and further away.”	“I took a couple of steps towards it, but the currents kept pushing the boat further and further away.”	Deletion
2	“I plunged into the ocean and I knew I had overcome my fear.”	“I plunged into the ocean and swam back to shore. As my father proudly looked on, I knew I had overcome my fear.”	Sentence combination
3	“I dived and swam back to shore.”	“I plunged into the ocean and swam back to shore.”	Paraphrase
4	“I was so traumatized.”	“In the days that followed, I was so traumatized that I would not go near the water.”	T.S.S (Beginning); Deletion
5	“He frantically searching for my body.”	“He repeatedly dived under the water, frantically searching for my body.”	T.S.S (End); Deletion
6	“I kicked hard, trying to remain above the surface.”	“Panic-stricken, I paddled and kicked hard, trying to remain above the surface.”	T.S.S (Title); Deletion
7	“My father was worried that the incident would scare me for life”	“My father was worried that the incident would scare me for life.”	Copy-verbatim
8	“My father plunged and swam as hard as he could to the spot where I had gone under and frantically searching for my body.”	“He dived in and swam as hard as he could to the spot where I had gone under. He repeatedly dived under the water, frantically searching for my body.”	Deletion; Sentence Combination; T.S.S (End); T.S.S (Title); Paraphrase.

Each strategy must have a unique or specific characteristic which can be used to identify the strategies. The steps to identify the characteristics of each strategy are explained as follows.

## Heuristic Rules for the Identification of Summarizing Strategies

### Deletion strategy

The main role of deletion strategy is to remove unimportant words or phrase from a sentence. It aims to delete phrase from the sentence if it is irrelevant to the main idea. To identify the deletion strategy, we use the following four rules:

#### Sentence length

It indicates the number of words in a sentence. The main task of deletion strategy is to eliminate unimportant information such as stop–words, explanations and examples from a sentence. Hence, the length of summary sentence in the summary text is always shorter than the corresponding sentence in source text. However, given two sentences, a summary sentence and the original sentence, let *S*_*s*_ be a summary sentence, *O*_*s*_ an original sentence, Len (*O*_*s*_) denotes the length of sentence *O*_*s*_ while Len (*O*_*s*_) denotes the length of sentence *S*_*s*_. The first rule for deletion strategy is as follows:

$$\text{Length}\;(S_{s})\;\text{is less than Length}\;(O_{s}).$$ (1)

Even though the deletion strategy removes some phrases from a sentence, it should keep the meaning of original sentence in new produced sentence. Hence, two additional rules should be considered. The following rules were also considered in order to identify deletion strategy:

#### Word overlapping

It considers the set of words (only non-stop words) occurring in both sentences. Given two sentences, let S_summary_ = {W_1_,W_2_, ⋯W_N_} be a sentence of summary text, where *N* is the number of words in the sentence S_summary_, S_original_ = {W_1_,W_2_, ⋯W_M_} is a sentence of original text, where *M* is the number of words in sentence *S*_*original*_. However, for each word from sentence *S*_*summary*_, the same word or the synonym word must be restated in sentence *S*_*original*_. Hence, the following statement can be made:

$$\forall \;W\;\in \;{\text{S}}_{summary}|\;W_{O}\;\in \;{\text{S}}_{original}$$ (2)

Where, *W* is a word of *S*_*summary*_ and *W*_*o*_ can be either a similar word or synonymous word.

#### Syntactic composition

It checks whether the syntactic composition of two sentences is equal. For example, given two sentences:

$${\text{S}}_{\text{original}}=\text{He}/\;\left(A\right)\;\text{repeatedly dived under the water, frantically searching}/\;\left(B\right)\;\text{for my body}/\;\left(C\right).$$

$${\text{S}}_{\text{summary}}=\text{He}\;/\;\left(A\right)\;\text{frantically searching}\;/\;\left(B\right)\;\text{for my body}\;/\;\left(C\right).$$

Suppose we select three words from sentence *S*_*summary*_; *A*, *B* and *C*. If the word *B* occurred after *A* and the word *C* occurred after *B*, this composition should occur in sentence *S*_*original*_. It means the word *B* must appear after word *A* and the word *C* must appear after word *B* in the *S*_*original*_ sentence. Thus, the following statement can be made:

$$\forall \;\text{A},\text{B},\text{C}\in {\text{S}}_{\text{summary}}\;:((\text{A}\;{\text{S}}_{\text{s}}\text{B}\;\wedge \;\text{B}\;{\text{S}}_{\text{s}}\text{C})\in {\text{S}}_{\text{summary}})\Rightarrow (({\text{A S}}_{\text{o}}\;\text{B}\;\wedge \;{\text{B S}}_{\text{o}}\;\text{C})\in {\text{S}}_{\text{original}})$$ (3)

Where,

*A S*_*s*_ *B*: *B* appears after *A* in sentence *S*_*summary*_.

*B S*_*s*_ *C*: *C* appears after *B* in sentence *S*_*summary*_.

*A S*_*o*_ *B*: *B* appears after *A* in sentence *S*_*original*_.

*B S*_*o*_ *C*: *C* appears after *B* in sentence *S*_*original*_.

Besides these rules for identifying the deletion strategy, in this study we also consider the similarity measure between two sentences as a rule to identify the deletion strategy. The similarity measure between two sentences is computed based on the semantic similarity and syntactic similarity between two sentences. We used Eqs (16) and (17) to calculate similarity measure between two sentences.

**Fig 1:**
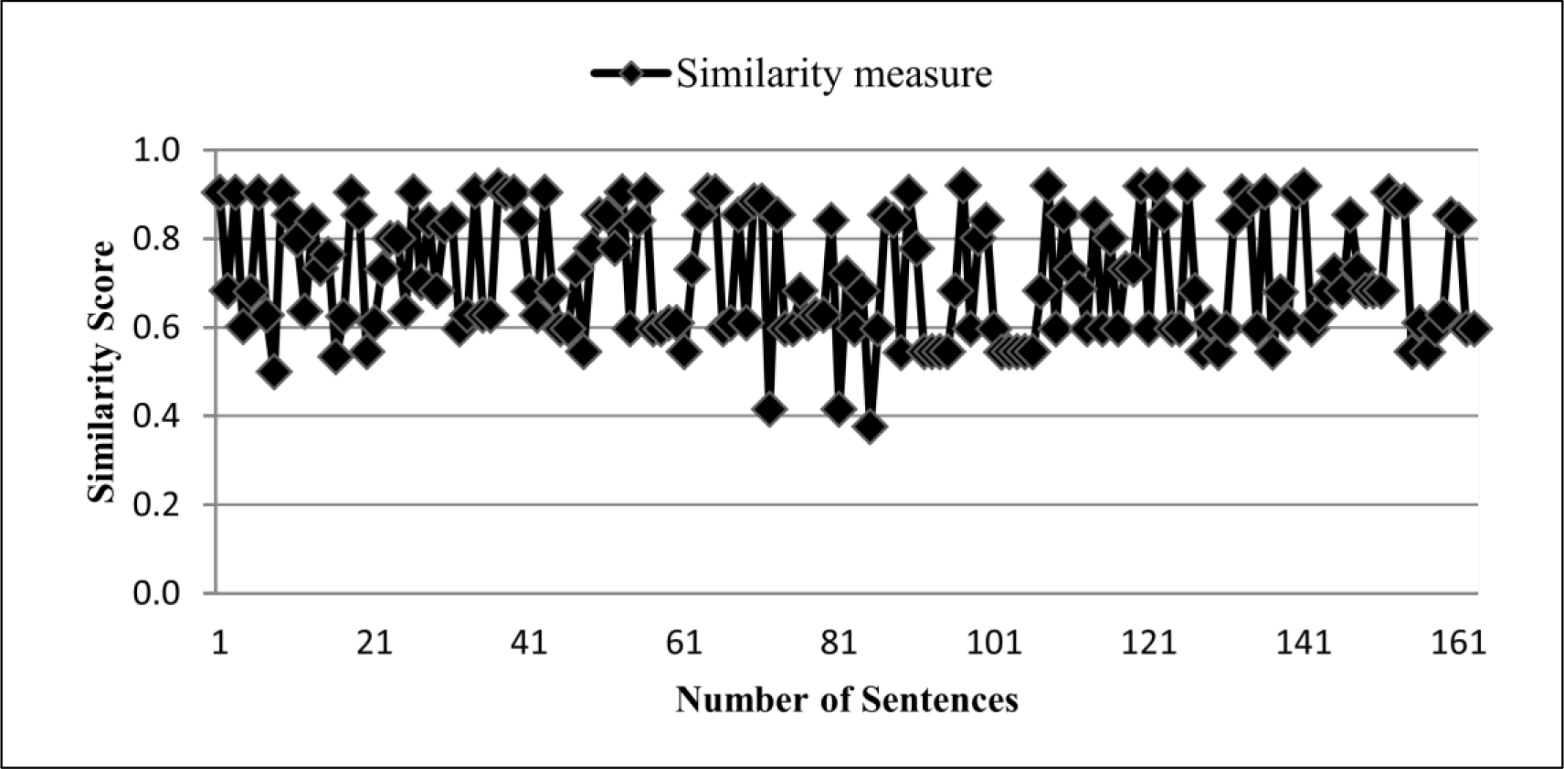
Sentence similarity measure in Deletion strategy.

In this study we collected 163 summary sentences produced by deletion strategy and their corresponding sentences from the source text. We then calculated the similarity measure between the sentence pairs by using Eqs (16)–(20). Fig 1 presents the results obtained in this study. Based on the analysis of the results, we found that the similarity measure between two sentences in deletion strategy was between 0 and 1, as shown in Fig 1. Thus, the following statement can be used as the fourth rule to identify deletion strategy:

$${\mathrm{Similarity}}_{\text{sentences}}\;\left({\text{S}}_{1},{\text{S}}_{2}\right)\;\text{is less than}\;1$$ (4)

From this study, we also found that in deletion strategy, only one sentence from the original text was used to create a summary sentence. Hence, we also consider this feature to identify deletion strategy. So, if *N* is the number of sentences that have been used for creating a summary sentence, then in deletion strategy we have the following statement:

The number of sentence(N)is equal to1. (5)

### Topic Sentence Selection (TSS) Strategy

The main objective of this strategy is to determine a sentence from a paragraph, which represents the main idea of the paragraph. To identify topic sentence selection strategy, we consider 4 methods which are key method, location method, cue method and title method. The methods are explained as follows.

#### Location method

This method assumes that sentences at the beginning as well as at the end of a document or a paragraph indicate the important information.

**Fig 2:**
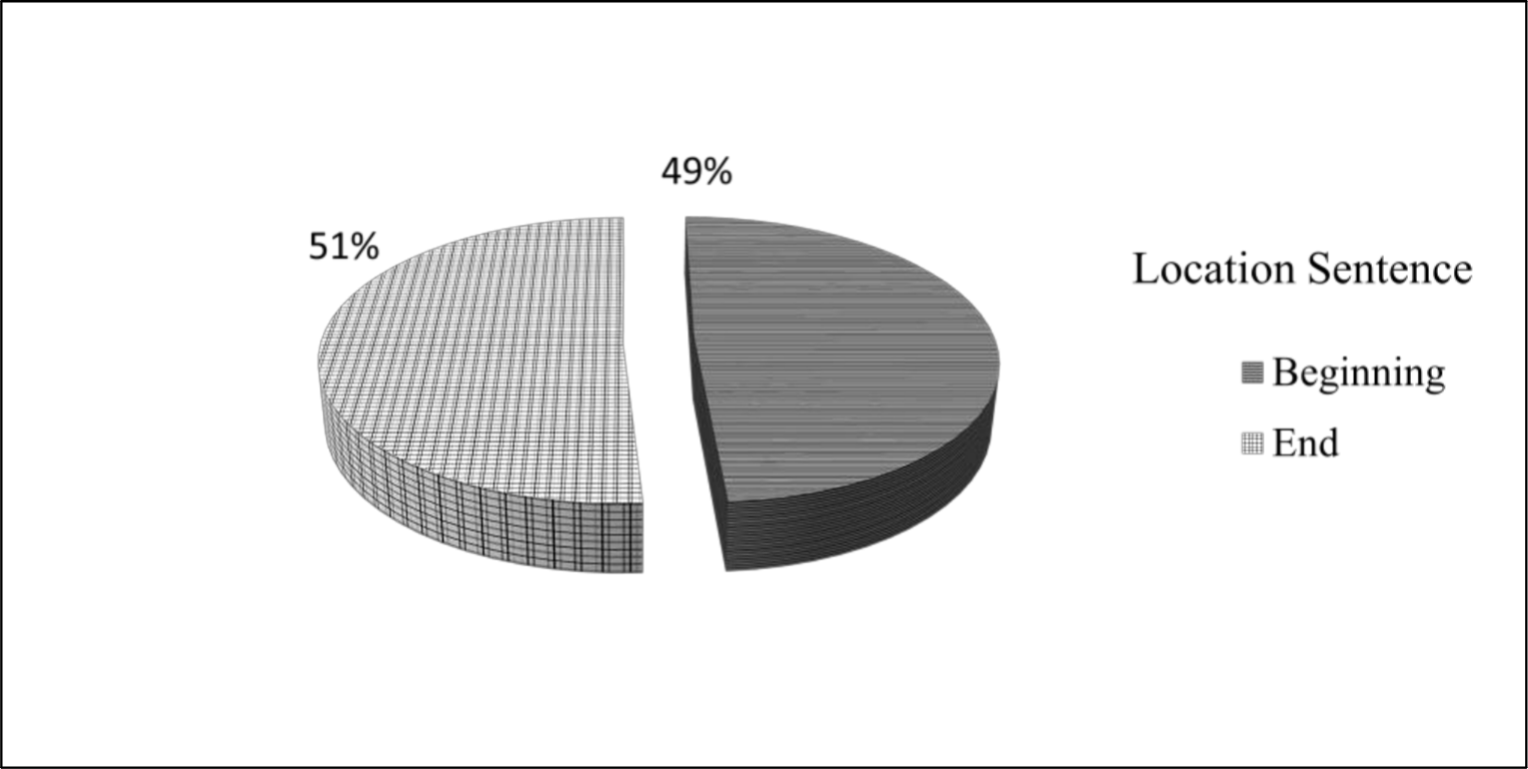
Use of Location Method amongst 560 sentences.

In this study, we investigated the use of location method to produce a summary sentence. For this purpose, we examined 560 summary sentences. We found that topic sentences tend to appear at the beginning or at the end of a paragraph. As shown in Fig 2, 49% and 51% of the topic sentences appeared at the beginning and the end of paragraphs, respectively. These findings are in agreement with the previous studies of Fattah and Ren [21] and Bawakid and Oussalah [27].

The following steps are used to identify topic sentence selection using location method:

1. Select all sentences from the source text that appeared at the beginning or at the end of a paragraph.
2. Add the selected sentences from step 1 to *Sentence Location List (SLL)*.
3. For each summary sentence, find the corresponding sentence from the source text. Let *S*_*summary*_ be a sentence of summary text, while *S*_*original*_ is a corresponding sentence of the original text that is used to produce the sentence *S*_summary._
4. Check the following statement to identify topic sentence selection:

$$\text{F}(\text{X})=\left\{ \begin{array}{l} TSS=1,X\;\in \;SLL\\ TSS=0,X\;\notin \;SLL \end{array}\right.$$ (6)

Where *X* indicates the sentence *S*_*original*_.

#### Key word method

**Fig 3:**
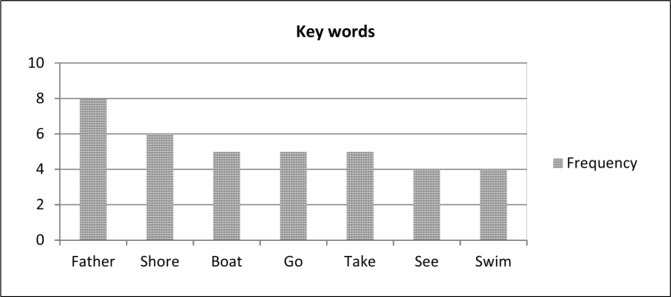
Frequency of keywords.

The assumption made by key word method is that the important sentences of a source text include one or more of key words. Key words are non-stop words, which occur frequently in the source text. We used term frequency (*Tf*) methods to identify words with high frequency in the source text, and then the words with high frequency were selected as the keywords. In this study, words with high frequency are shown in Fig 3.

**Fig 4:**
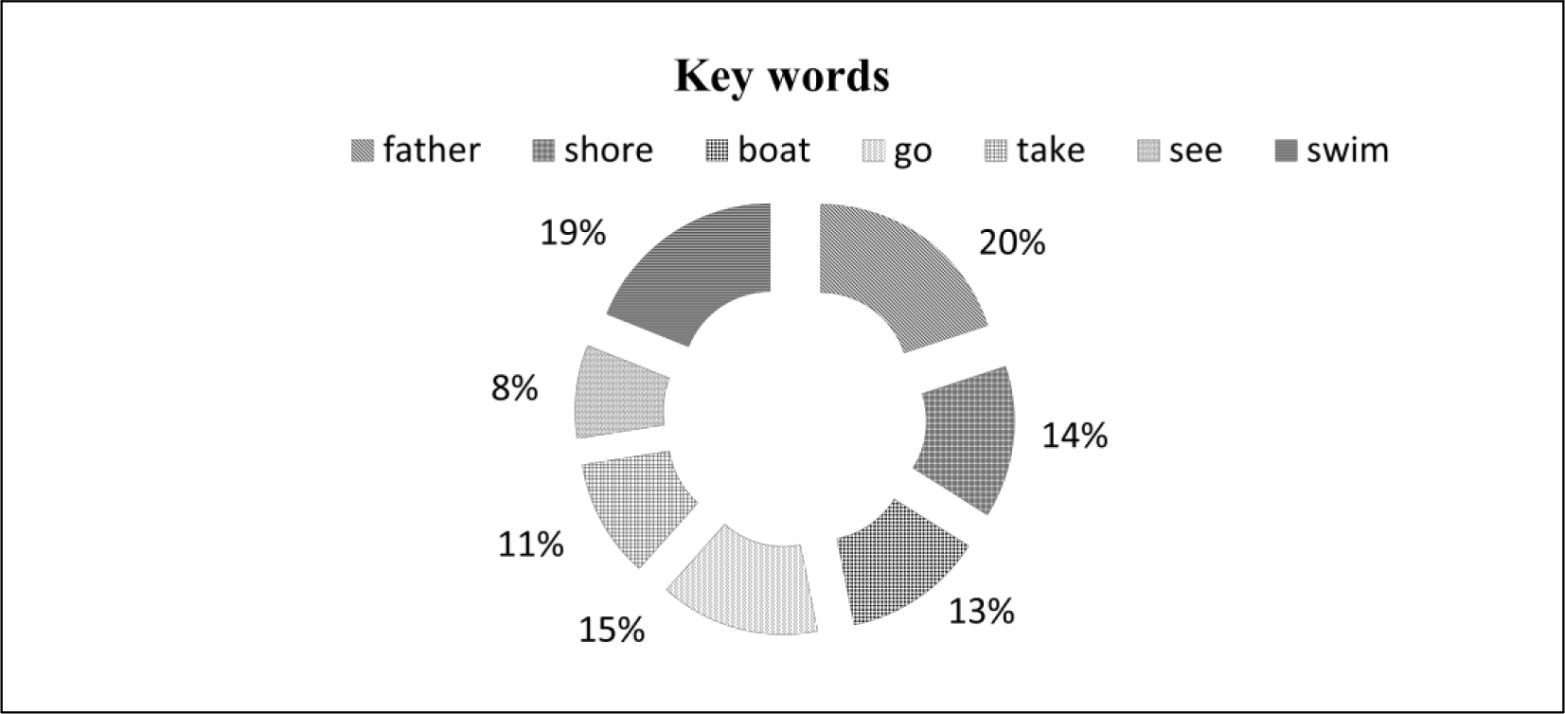
Use of keywords in summaries.

In this study, we identified the sentences from the source text that are used to produce summary sentences which consist of these key words. From the analysis of these sentences, we found that all of these sentences include keywords. The result of our study is presented in Fig 4. It shows the percentage use of keywords in summarises for identifying topic sentence selection strategy.

The following steps are used to identify topic sentence selection using keyword method:

1. Remove all stop-words from the source sentences.
2. Identify the frequency of each word of the source text.
3. Select top *N* words with high frequency, and then add them to *Keywords List (KL)*.
4. Find the corresponding sentence from source text for each summary sentence. Let *S*_*summary*_ be a summary sentence, and *S*_*original*_ be a corresponding sentence of the original text that is used to produce the sentence *S*_*summary*_.
5. Check the following statement to identify topic sentence selection:

$$\text{F}\left(\text{Y}\right)=\{\begin{array}{l} TSS=1,Y\;\in \;KL\\ TSS=0,Y\;\notin \;KL \end{array}$$ (7)

Where *Y* indicates a word of S_original_.

#### Title Method

**Fig 5:**
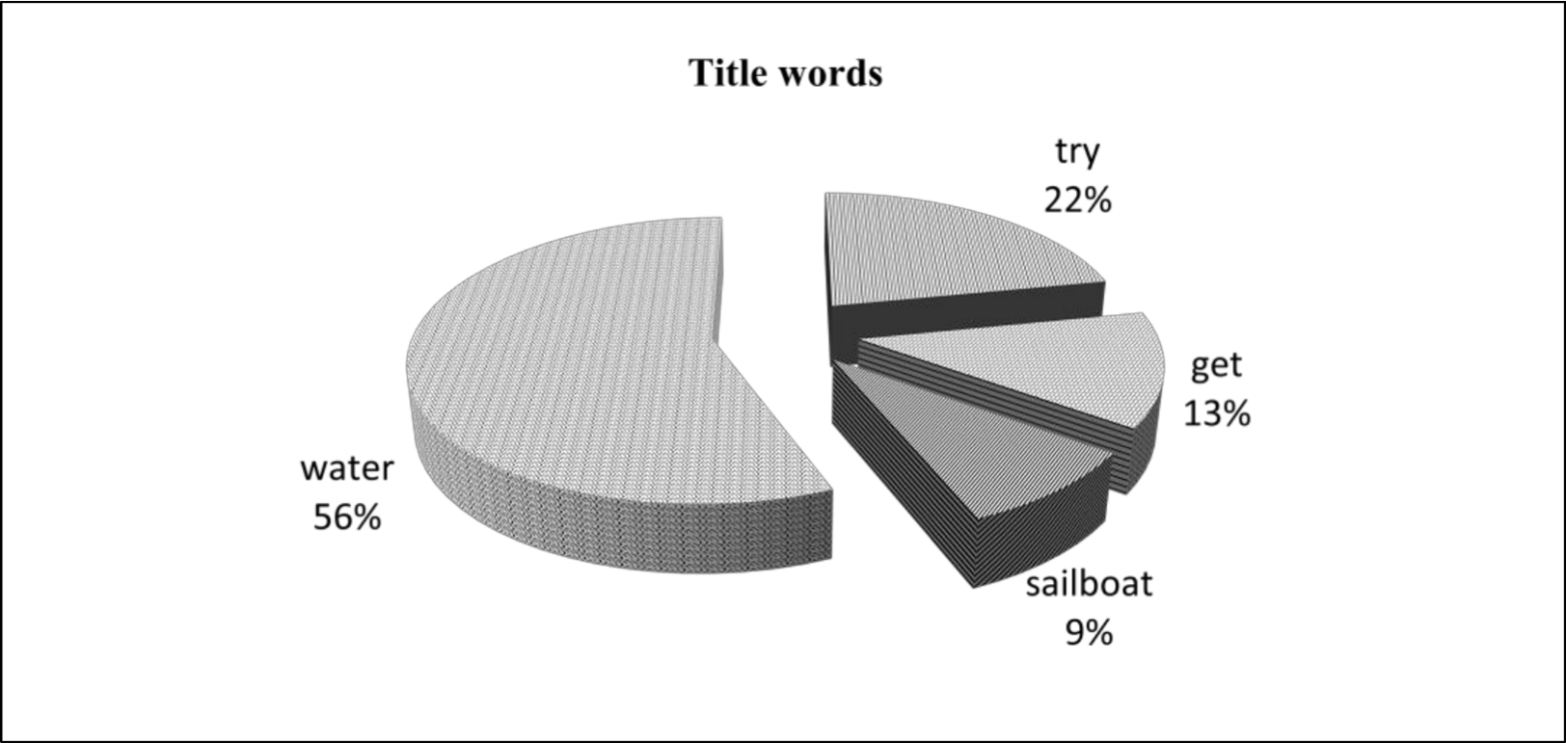
Use of Title words amongst summaries.

In title method, if a sentence of the original text contains one or more of the words that appeared in the title, the sentences can be considered as a topic sentence. In this study, we identified the sentences from the source text that are used to produce summary sentences which consist of title words. The result of our study is presented in Fig 5. It shows the percentage use of each word from text title that has been used to select an important sentence in topic sentence selection strategy.

The following steps are used to identify topic sentence selection using title method:

1. Add all words (non-stop words) to *Title List (TL)*.
2. Find the corresponding sentence from source text for each sentence of summary text. Let *S*_*summary*_ be a sentence of summary text, *S*_*original*_ be a corresponding sentence of the original text that is used to produce the sentence *S*_*summary*_.
3. Check out the following statement for identifying topic sentence selection:

$$F\left(Z\right)=\{\begin{array}{c} TSS=1,Z\;\in \;TL\\ TSS=0,Z\;\notin \;TL \end{array}$$ (8)

Where *Z* indicates a word of *S*_*original*_.

#### Cue method

**Fig 6:**
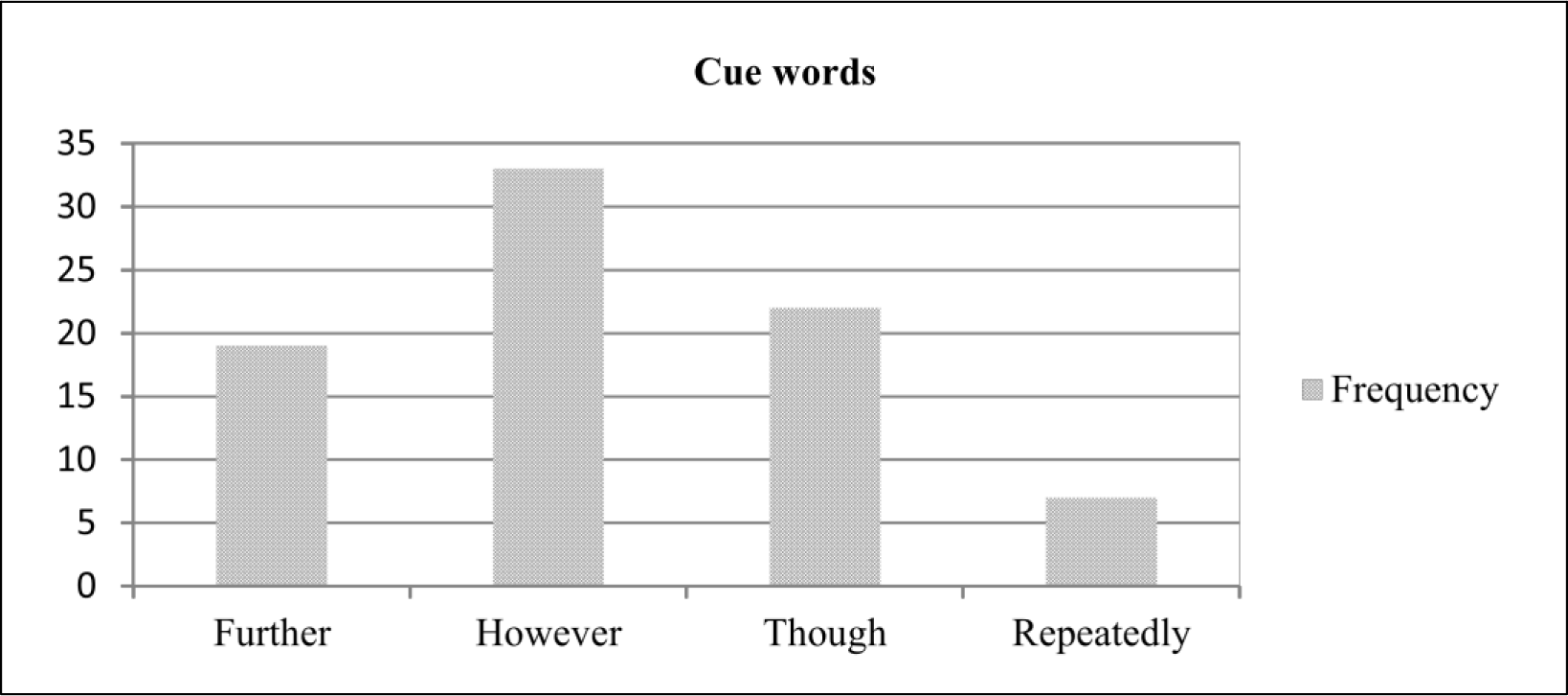
Frequency of cue words in summaries.

Cue method includes cue words or phrases such as “*in conclusion”*, “*in this paper”*, “*our investigation has shown”*, and “*a major result is”*. The presence of these words in a sentence indicates the important information in the source text. These cue words are context dependent. However, due to the existence of different type of text, such as scientific article and newspaper article, it is difficult to collect these cue word as a unit list. Hence, since discourse markers can be used as an indicator of important content in a text and are more generic, a list of cue words has been built using discourse markers. In this study, we found some discourse markers that were used to indicate the significance of a sentence. Fig 6 presents some of these cue words.

The following steps are used to identify topic sentence selection using cue method:

1. Construct a *Cue word list (CWL)* using the discourse marker.
2. Find the corresponding sentence from source text for each summary sentence. Let *S*_*summary*_ be a summary sentence, *S*_*original*_ be a corresponding sentence of the original text that is used to produce the sentence *S*_*summary*_.
3. Check the following statement to identify topic sentence selection:

$$\text{F}\left(\text{CWL}\right)=\{\begin{array}{c} TSS=1,K\;\in \;CWL\\ TSS=0,K\;\notin \;CWL \end{array}$$ (9)

Where *CWL* indicates a word of *S*_*original*_.

### Paraphrasing strategy

Paraphrase strategy is a way to replace a word in source sentence with a synonym or similar word in summary sentence. For example, given two sentences (A: “*I plunged into the ocean and swam back to shore*.”) and (B: “*I dived into the ocean and swam back to shore*.*”*). The word ‘*plunged*’ in sentence *A* was replaced by a synonym word “*dived”*.

The following steps are used to identify paraphrasing strategy:

1. Let S_summary_ = {W_1_,W_2_, ⋯W_N_} be a summary sentence and S_original_ = {W_1_,W_2_, ⋯W_M_} be a corresponding sentence of the original text that is used to produce the sentence *S*_*summary*_, where *M and N* are the number of words.
2. Get the root of each word of *S*_*original*_ using WordNet, and then add to *Array Root (AR)*.
3. Get the synonym of each word of *S*_*original*_ using WordNet, and then add to *Array Synonym (AS)*.
4. For each word of *S*_*summary*_, get the root of word using the WordNet, Let *RW* be the root of the word, then check out the following conditions:.
  - If *RW* was in *AR*, then set paraphrase strategy to “0”, then jump to step 4; otherwise continue the following step.
  - If *RW* was in *AS*, then set paraphrase to “1”; Stop the current loop; Otherwise jump to (iii);
  - Calculate the semantic similarity between *RW* and all word from *S*_*original*_ using Eqs (16) and (17).
  - If there is a similar value, then set paraphrase to “1”; Stop the current loop; Otherwise jump to 4;

### Sentence Combination Strategy

The main objective of the sentence combination strategy is to combine one or more sentences from the source text to construct a summary sentence. It uses conjunction words such as *and*, *or*, *so* and etc., to merge sentences into a single sentence. In this study, we examined two features such as the number of source sentences combined in each summary sentence and the similarity measure between two sentences, summary sentence and the corresponding sentence of the source text. For this purpose, we collected 105 summary sentences produced using sentence combination strategy.

**Fig 7:**
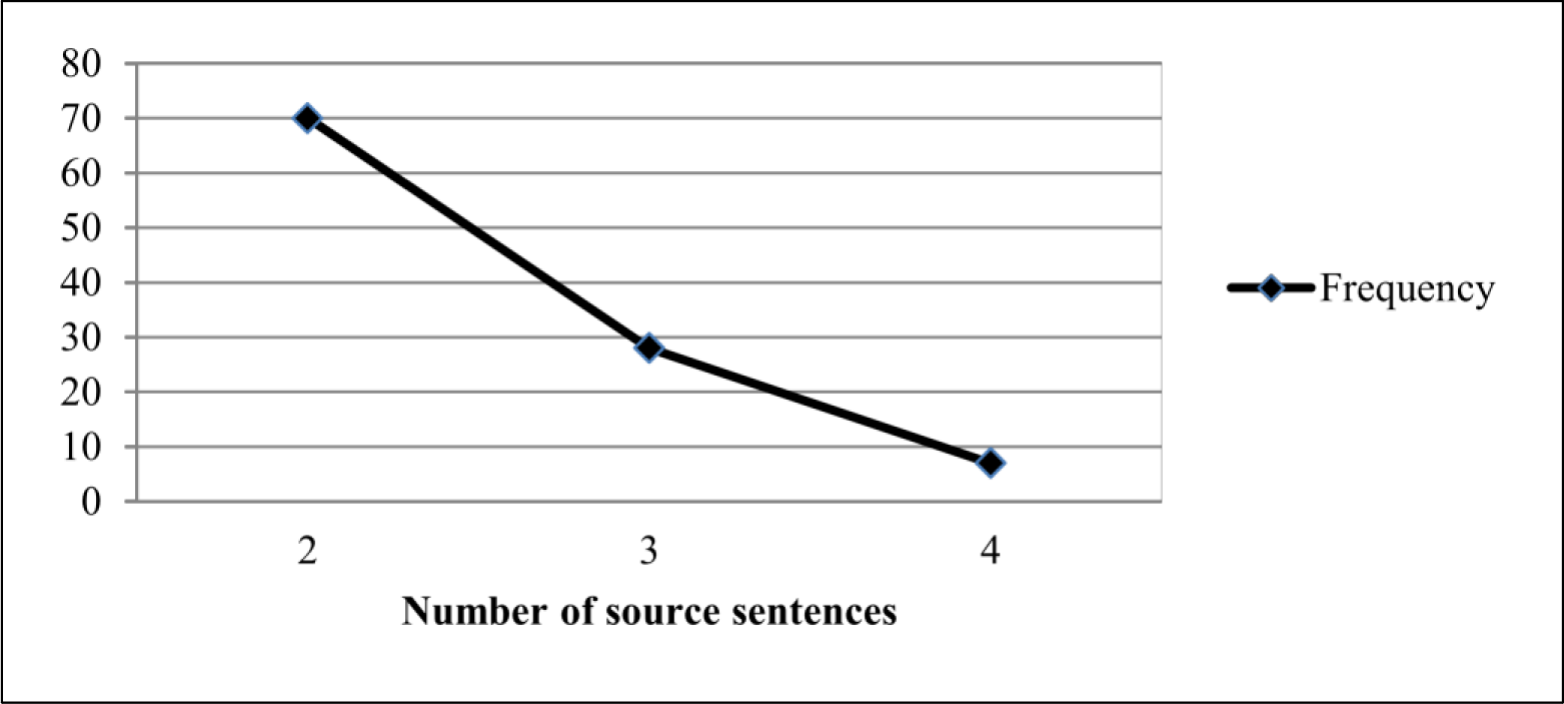
Number of source sentences combined in each summary sentence.

To examine how many sentences are normally merged in a summary sentence, we analysed the number of source sentences that have been used to create a summary sentence. From the analysis, we found that most summary sentences are generated from two or three sentences of the source text. Fig 7 presents the number of source sentences included in summary sentences. As we can see in Fig 7, out of 105 summary sentences created using sentence combination strategy, 70 summary sentences were usually a combination of two source sentences, 28 summary sentences were produced from 3 source sentences and 7 summary sentences were generated by 4 source sentences. As a result from this study, the following statement can be used as a rule to identify sentence combination strategy:

The number of sentences(N)is greater than1. (10)

Where, *N* is the number of source sentences which have been used to produce a summary sentence.

Besides the aforementioned rules for identifying sentence combination, in this study we also consider *the similarity measure between a summary sentence and the number of sentences from the source text involved in summary sentence*, as a rule to identify this strategy. The similarity measure is computed based on the semantic similarity and syntactic similarity between two sentences.

The following steps are used to calculate the similarity measure in sentence combination strategy:

1. Given a Summary Sentence (SS) = {P_1_, P_2_⋯P_N_}, where *P*_*1*_, *P*_*2*_ and *P*_*N*_ are phrases from summary sentence that came from *T*_*1*_, *T*_*2*_, and *T*_*M*_ respectively. *T*_*1*_, *T*_*2*_, and *T*_*M*_ are source sentences that are used to produce the summary sentence.
2. Calculate the similarity measure between each pair of sentences, such as (T_1_,SS), (T_2_,SS)⋯, and (T_M_,SS) using the following steps:
  - Create a “word Set”.
  - Calculate semantic similarity between two sentences using Eq 18.
  - Calculate syntactic similarity between two sentences using Eq 19.
  - Calculate similarity measure between two sentences based on the semantic similarity and syntactic similarity using Eq 20.
3. Calculate the average similarity measure between sentences using the following equation:

$$Ave_{\text{similarity measure}}=\;\frac{\sum_{i=1}^{M}\;Sim\left(T_{i},S_{summary}\right)}{M}$$ (11)

Where, *M* is the number of source sentences.

**Fig 8:**
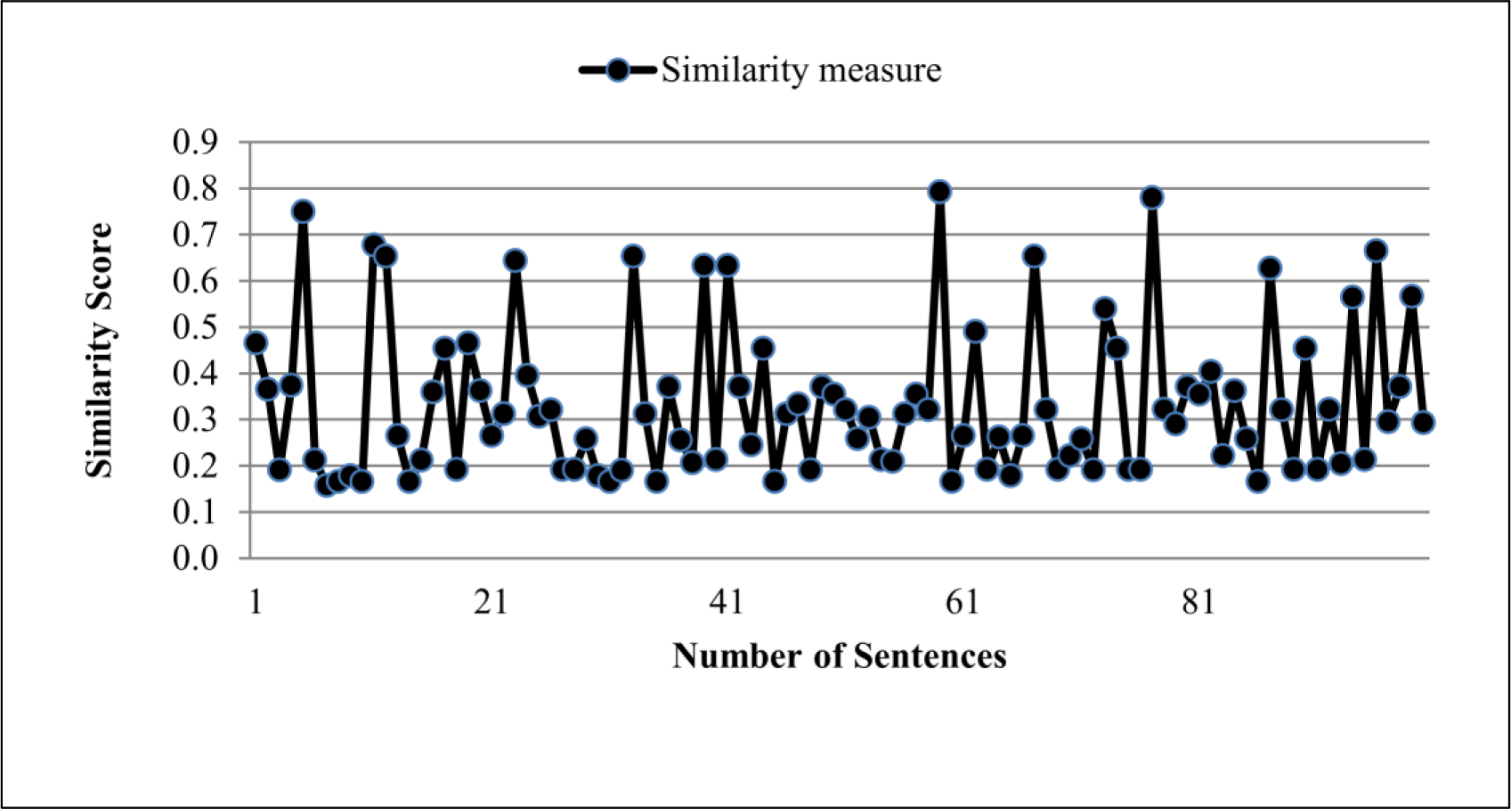
Sentence similarity measure in Sentence combination strategy.

In this study, we collected 100 summary sentences produced by sentence combination strategy and the corresponding sentences from the source text. Then, we calculate the similarity measure between sentence pairs by using Eqs (16) and (17). From the analysis of the results, we found that the similarity measure between sentences in sentence combination strategy is between 0 and 1, as shown in Fig 8. Therefore, the following statement can be used as a rule to identify sentence combination strategy:

$$Ave_{\text{similarity measure}}=\;\frac{\sum_{i=1}^{M}\;Sim\left(T_{i},S_{summary}\right)}{M}<1$$ (12)

### Copy–verbatim

In the copy–verbatim process, a summary sentence is created from the source sentence without any changes. This strategy is not part of the summarizing strategies but it is one of the common strategies that is used by students. To identify the copy–verbatim strategy, we use the following three rules:

#### Sentence length

Sentence length, contains the number of words in a sentence. The main task of copy–verbatim strategy is to produce a summary sentence using a source sentence without any changes. Therefore, the length of summary sentence in summary text is always equal to the length of the corresponding sentence in the source text. Given two sentences, summary sentence and original sentence, let *S*_*s*_ be a summary sentence, *O*_*s*_ be an original sentence, let Len (*O*_*s*_) denote the length of sentence *O*_*s*_ and Len (*S*_*s*_) denote the length of sentence *S*_*s*_. The first rule can have the following statement:

$$\text{The Length}\;(S_{S})\;\text{is equal to the Length}\;(O_{S}).$$ (13)

#### Similarity measure between sentences

In this study, to identify copy–verbatim strategy, we also consider the similarity measure between two sentences as a rule to identify this strategy. The following steps are used to calculate similarity measure between two sentences:

1. *Create a “word set”*.
  1. Calculate semantic similarity between two sentences using Eq 18.
  2. Calculate syntactic similarity between two sentences using Eq 19.
  3. Calculate similarity measure between two sentences based on the semantic similarity and syntactic similarity using Eq 20.

We collected 80 summary sentences produced by copy–verbatim strategy and the corresponding sentences from the source text. Then, we calculated the similarity measure between sentence pairs. We found that the similarity measure between two sentences in copy–verbatim strategy is bigger than 0 and equal to 1. Thus, the following statement can be used as a second rule to identify copy–verbatim strategy:

$$\text{The}\;Similarity_{\text{sentences}}\;\left({\text{S}}_{1}\;,{\text{S}}_{2}\right)\;\text{is equal to}\;1.$$ (14)

#### Total number of sentences

In copy–verbatim strategy we detected only one sentence from the original text used to produce a summary sentence. Hence, we also consider this feature to identify this strategy. So, if *N* is the number of sentences that have been used to produce a summary sentence, then in copy–verbatim strategy we have the following statement that can be used as a third rule to identify strategy:

The number of sentence(N)is equal to1. (15)

The summarizing strategies found from the decomposition of summary text were analyzed and formalized into a set of heuristic rules on how to identify the summarizing strategies. These rules are given in Table 3.

**Table 3:** The rules to identify summarizing strategies and methods.

Summarizing Strategies	Heuristic rules to identify summarizing strategies
Deletion	1. Words of summary sentence are found in source sentence.
	2. The syntactic composition of the words in the summary sentence and in the corresponding source sentence is the same.
	3. The number of words in summary sentence is less than the number of words in the corresponding source sentence.
	4. *TN* = 1 && Sim (S_r,_ S_s_**) <** 1
Sentence combination	*1*. The summary sentence contains a combination of phrases from two or more sentences in the original text.
	2. TN > 1 && (∑_(i = 1)_^TN^ Sim (S_r,_ S_s_)) / TN < 1
Paraphrase	1. A word in the source sentence is replaced with a synonym word in the summary sentence.
Topic Sentence Selection (TSS)	A summary sentence is created by TSS, if it used:
	1. Title method: The sentence includes one or more of Title words.
	2. Location method: The sentence should be the first or last sentence of paragraph.
	3. Cue method: The sentence includes one or more of cue phrases.
	4. Keyword method: The sentence includes one or more of Key words.
Copy–verbatim	1. All words of summary sentence are found in source sentence.
	2. The position of the words in the summary sentence and in the corresponding source sentence is the same.
	3. The number of words in summary sentence is equal to the number of words in the source sentence.
	4. *TN* = 1 && Sim (S_r,_ S_s_) **=** 1

Where,

S_s_: denotes a summary sentence.

RS = {S_1_,…S_n_}: denotes the Relevant Sentences (*RS*) that are used to produce the *S*_S._

TN: denotes the total number of sentences in *RS*.

S_r_: denotes a sentence of *RS*.

Sim (S_r,_ S_S_): denotes the sentence similarity measurement, Eq (20).

## Related Works

There exists a large research on how the computer can help writing summaries: either by carrying out summarization or by evaluating students’ summaries. However, computer models of the methods employed by instructors to evaluate students’ summaries are yet lacking. An implementation of these models is more difficult, since many complicated goals must be considered to implement these models: those have to identify the important information or main idea from a source text (i.e., sentences/paragraph), then to perform a summarizing strategy (i.e., what kind of summarizing strategies to accomplish on these sentences/paragraph). Despite of the difficulty to implement these models, recently, researchers have developed a few systems for summary assessment.

In this section, first, the summary assessment systems those focus on content and style are introduced. Then, the summary assessment systems those focus on identifying summarizing strategies are introduced.

Laburpen Ebaluaka Automatikoa (LEA)[28], which is based on Latent Semantic Analysis (LSA) and cosine similarity measure, has been proposed to evaluate the output of the summarizing process. It is designed for both teachers and students, and enables teachers to examine the student-written summary, as well as allows students to produce a summary text using their own words. The summaries are evaluated based on certain features, such as cohesion, coherence, the use of language, and the adequacy of the summary.

Summary Street [29], which is based on LSA, is a computer-based assessment system that is used to evaluate the content of the summary text. Summary Street ranks a student-written summary by comparing the summary text and source text. It creates an environment to give appropriate feedback to the students, such as content coverage, length, redundancy and plagiarism.

Lin [30] proposed an automatic summary assessment system named Recall-Oriented Understudy for Gisting Evaluation. It is used to assess the quality of the summary text. The current system includes various automatic assessment approaches, such as ROUGE-N, ROUGE-L and ROUGE-S. ROUGE-N compares two summaries based on the total number of matches. ROUGE-L calculates the similarity between a reference text and a candidate’s text based on the Longest Common Subsequence (LCS). ROUGE-S (Skip-Bigram Co-Occurrence): skip-bigram is any pair of words in their sentence order, allowing for arbitrary gaps.

FRESA (Framework for Evaluating Summaries Automatically) [31], which is based on Jensen-Shannon divergence and ROUGE is a framework that is used to evaluate the multilingual summarization without Human references. It used the Rouge package such as uni-grams, bigrams, and the skip bi-grams with maximum skip distance of 4 (ROUGE-1, ROUGE-2 and ROUGE-SU4), to compute various statistics.

Mohler, Bunescu [32] introduced an Answer Grading System, which combines a graph alignment model and a text similarity model. This system aims to improve the existing approaches that automatically assign a grade to an answer provided by a student, using the dependency parse structure of a text and machine learning techniques. The current system uses the Stanford Dependency Parser [33] to create the dependency graphs for both the student (*A*_*1*_) and teacher (*A*_*2*_) answers. For each node in the student’s dependency graph the system computes a similarity score for each node in the teacher’s dependency graph using a set of lexical, semantic, and syntactic features. The similarity scores are used to weight the edges that connect the nodes in *A*_*1*_ on one side and the nodes in *A*_*2*_ on the other. The system then applies the Hungarian algorithm to determine both an optimal matching and the score associated with such a matching for the answer pair. Finally, the system produces a total grade based on the alignment scores and semantic similarity measures.

Although previous systems [28, 29, 30, 31] have developed to assess summary writing, they focus on the content of the summary. A few summarization assessment systems have been developed to identify the summarizing strategies used by students in writing a summary. To the best of our knowledge, there are two systems which have been developed for summary assessment. We explain each of them as follows.

Modelling summarization assessment strategies (MSAS)[14] based on LSA have been developed. This model is based on the identification of 5 types of strategies which are:

1. *Copy*, a sentence from a summary text is semantically very close to a sentence in a source text.
2. *Paraphrase*, a sentence from a summary text is close to only one sentence in a source text.
3. *Generalization*, a sentence from a summary text is close to several sentences in a source text.
4. *Construction*, if no sentences of the original text are close to the summary sentence but at least one of them is related.
5. *Off-the-subject*, if all sentences of the original text are not related to the summary sentence.

Using LSA and cosine similarity, each sentence from summary text is semantically compared with all sentences in a source text to identify the summarizing strategies. Three similarity thresholds have been used to create four categories: not enough similarity (cosine is less than 0.2), low similarity (cosine is greater than 0.2 and less than 0.5), good similarity (cosine is greater than 0.5 and less than 0.8), too high similarity (cosine is greater than 0.8). The comparison between each sentence from summary text and each sentence from source text results in a distribution of similarities among these four categories which lead to the identification of the student strategy.

Summary Sentence Decomposition Algorithm (SSDA) [15], which is based on word–position, has been proposed to identify the summarizing strategies used by students in summary writing. In this system, the summary text is syntactically compared with the source text to identify the summarizing strategies such as deletion, sentence combination and copy–verbatim. It does not use the semantic relationships between words in comparison between two sentences; hence, it cannot find summarizing strategies at the semantic level, such as paraphrasing, generalization, and invention.

## Focusing on Main Problem

**Fig 9:**
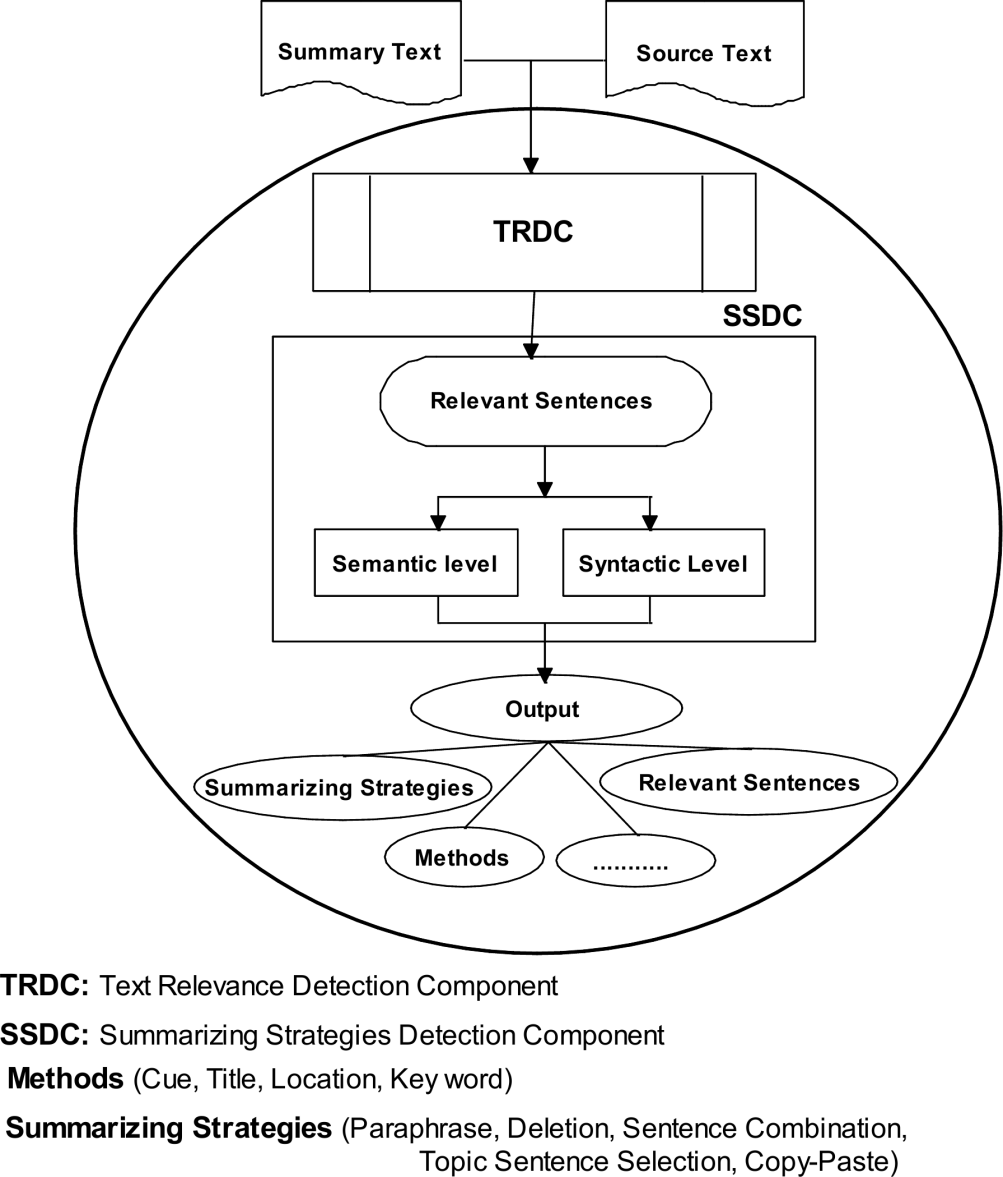
The processes of identifying summarizing strategies.

Conceptually, the process of identifying summarizing strategies involves two sub- processes as shown in Fig 9: 1) identifying the sentences from the source text that were used to create the summary sentences; and 2) identifying the summarizing strategies based on the sentences that have been identified in the first process. Before identifying the summarizing strategies, the Text Relevance Detection Component (TRDC) should be able to determine the relevant sentences from the source text, for each summary sentence. If the relevant sentences cannot be determined from the source text, no matter how well other components in the system perform, the summarizing strategies will not be identified. Therefore, the text relevance detection component is an important engine in identifying summarizing strategies. This module provides a list of sentences which will be analysed in further steps. These sentences are then further processed using a variety of techniques to identify the summarizing strategies has been used in summary writing.

In text relevance context, linguistic knowledge such as semantic relations between words and their syntactic composition, play key role in sentence understanding. This is particularly important in comparison between two sentences where a single word token was used as a basic lexical unit for comparison.

Syntactic information, such as word order, can provide useful information to distinguish the meaning of two sentences, when two sentences share the similar bag-of-words. For example, “*student helps teacher*” and “*teacher helps student*” will be judged as similar sentences because they have the same surface text. However, these sentences convey different meanings. On other hand, two sentences are considered to be similar if most of the words are the same or if they are a paraphrase of each other. However, it is not always the case that sentences with similar meaning necessarily share many similar words. Hence, semantic information such as semantic similarity between words and synonym words can provide useful information when two sentences have similar meaning, but they used different words in the sentences.

While both semantic information and syntactic information contribute in sentence understanding [34, 35, 36, 37, 38] the current systems that have been proposed to identify summarizing strategies did not use the combination of semantic relations between words and their syntactic composition to identify text relevancy. Obviously this drawback has a negative influence on the performance of the previous systems.

As shown in Fig 9, there are two levels of summarizing strategies, semantic and syntactic levels. The strategies in semantic levels include paraphrase, generalization, topic sentence selection and invention. The strategies in syntactic level include deletion, copy verbatim and sentence combination. A few systems have been proposed to identify summarizing strategies [14, 15]. However, these systems can identify strategies either in semantic level or in syntactic level. On the other hand, these systems did not use the combination of semantic and syntactic information to determine the relevant sentences from the source text, for each summary sentence. Obviously these disadvantages have a negative effect on the performance of current systems.

## ISSLK Algorithm

The ISSLK combines semantic information and syntactic information to identify relevant sentences and summarizing strategies. The ISSLK algorithm is developed to:

1. Determine whether a sentence in the summary text is from the original text. Let *S*_*s*_ represent a sentence of the summary text.
2. Identify all sentences from the original text that have relations with *S*_s._ Let *R*_*relations*_ include these sentences.
3. Identify all sentences from *R*_*relations*_ that are used to produce sentence *S*_s._ Let *P*_*Relevant Sentences*_ include these sentences.
4. Identify the summarizing strategies and methods used to produce a summary sentence using sentences from *P*_*Relevant Sentences*_.

This algorithm includes two sub-algorithms, which are:

### Sentences Relevance Identification Algorithm

The sentences relevance identification algorithm is a process for identifying sentences from the source text, which are used to produce a sentence in the summary text. It uses the combination of semantic similarity and syntactic similarity to identify these sentences. The steps to determine these sentences are presented in the *intermediate-processing stage*.

### Summarizing Strategies Identification Algorithm

After identifying the relevant sentences for each sentence of summary text, the summarizing strategies that have been used to produce a summary sentence are identified. This process involves the use of the rules, as shown in Table 3, in which the rules are transformed into an algorithm as presented in the *post-processing stage*.

**Fig 10:**
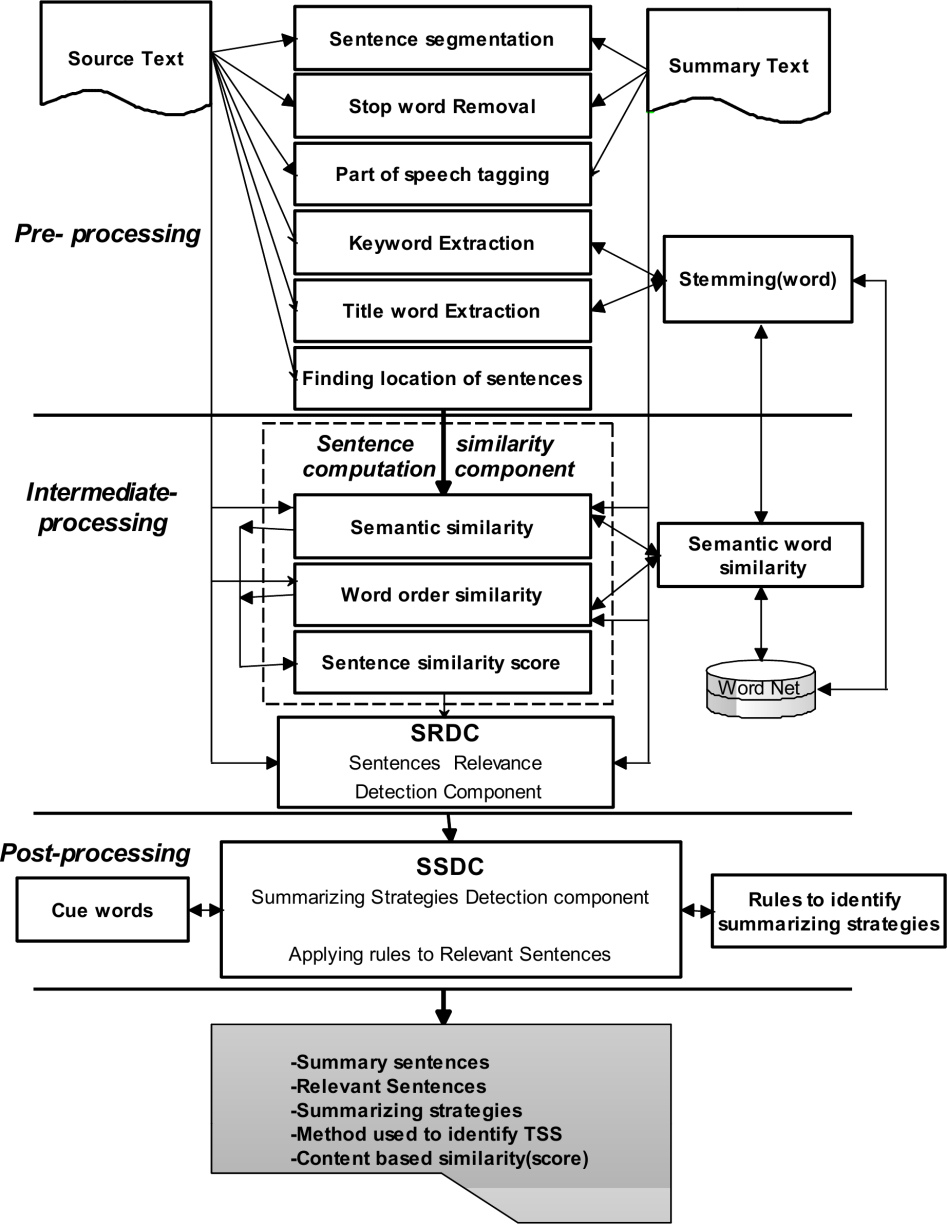
Overview of the development of the ISSLK.

Fig 10 displays the general architecture of the ISSLK algorithm, which consists of three main stages: a) Pre-processing, b) Intermediate-processing, and, c) Post-processing.

### Pre-processing

This stage aims to perform a basic linguistic analysis on both the source text and students' summaries. Thus, it prepares them for further processing. In order to perform this analysis, external tool and resource are used. The pre-processing module provides text pre-processing functions, such as sentence segmentation, tokenization, part-of-speech tagging, stemming, stop word removal, finding sentences location (FSL), keyword extraction (KE) and title word extraction (TWE). The FSL finds the location of each sentence in a source text and determines whether it is the first or the last sentence of a paragraph or document. The TWE extracts all the nouns and verbs from the title of a document. The KE uses the Term Frequency (TF) method to identify words with high frequency.

### Intermediate-processing

Intermediate processing is the core of the ISSLK algorithm and determines whether the summary sentence is generated from the source text, and, if so, identifies all the relevant sentences from the original text that are used to produce the summary sentence. To do so, the intermediate processing uses the Sentence Similarity Computation Component (SSCC) and Sentences Relevance Detection Component (SRDC). We describe each of them as follows:

#### Sentence Similarity Computation Component (SSCC)

**Fig 11:**
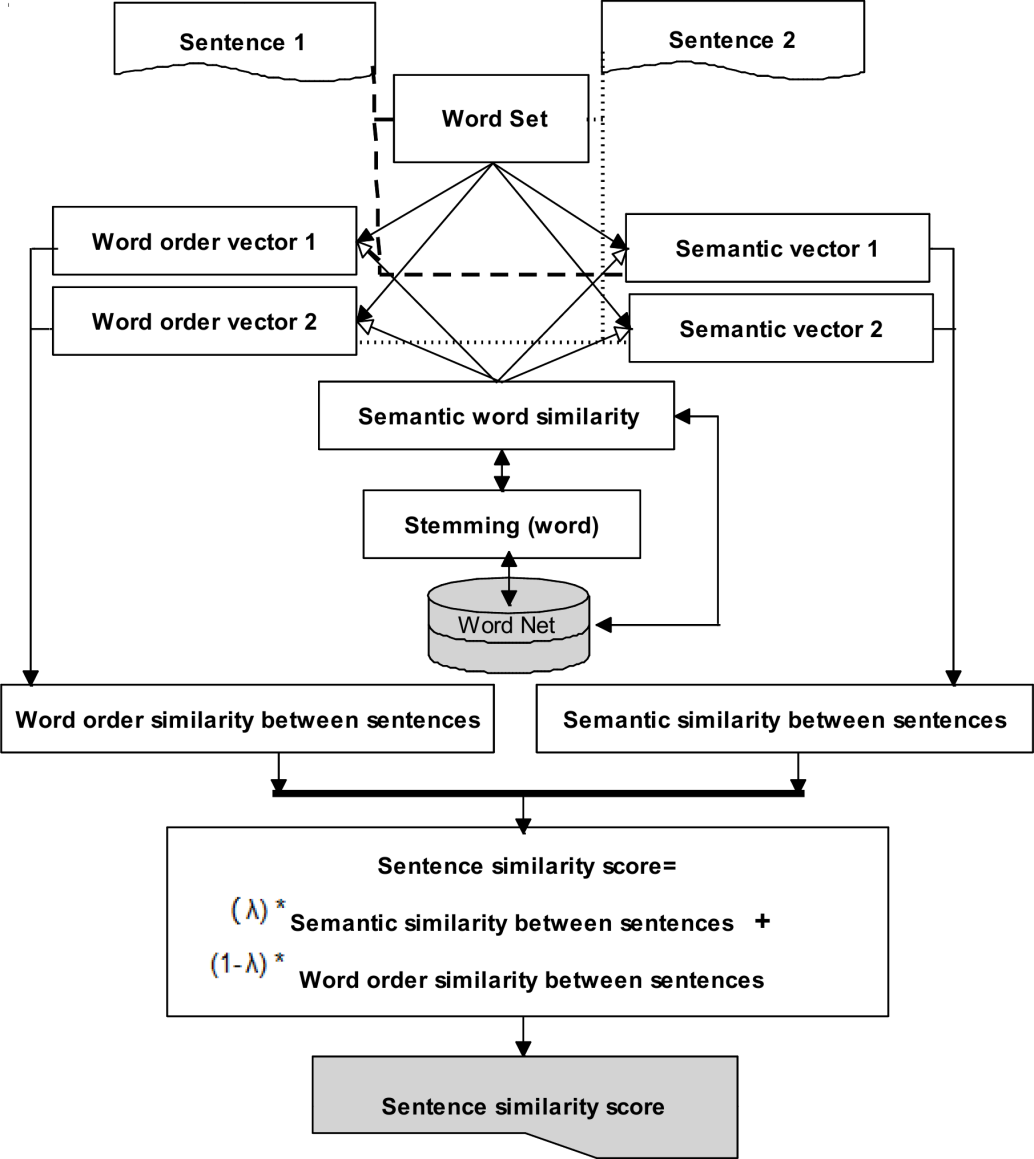
Sentence similarity computation model.

The sentence similarity computation component includes a computation model to calculate the sentence similarity measure. The Sentence Similarity Computation Model (SSCM) is presented in Fig 11. It shows the overall process of applying the semantic and syntactic information to determine the similarity measure between two sentences. The main task of SSCM is to identify all the sentences from the original text that have relations with a sentence of summary text. This model includes a few components, such as word set, semantic similarity between words, semantic similarity between sentences, syntactic similarity between sentences, and sentence similarity measurement. The task of each component is as follows:

The word set–Given two sentences *S*_*1*_ and *S*_*2*_, a “word set” is created using distinct words from the pair of sentences. Let WS = {W_1_, W_2_⋯W_N_} denote word set, where *N* is the number of distinct words in the word set. The word set between two sentences is obtained through certain steps as follows:

1. Two sentences are taken as input.
2. Using a loop for each word, *W*, from *S*_*1*_, certain tasks are undertaken, which include:
  - Determining the root of *W* (denoted by *RW*) using the WordNet.
  - if the *RW* appears in the *WS*, jumping to step 2 and continuing the loop using the next word from *S*_*1*_, otherwise, jumping to step iii;
  - If the *RW* does not appear in the *WS*, then assigning the *RW* to the *WS* and then jumping to step 2 to continue the loop using the next word from *S*_*1*_.
  - Conducting the same process for Sentence 2.

Semantic Similarity between Words (SSW)–Semantic word similarity [39, 40] plays an important role in this method. It is used to create a word order vector and semantic vector. The semantic similarity between two words is determined through these steps:

1. Two words, *W*_*1*_ and *W*_*2*_, are taken as input.
2. the root of each word is obtained using the lexical database, WordNet;
3. the synonym of each word is obtained using the WordNet;
4. the number of synonyms for each word is determined;
5. the Least Common Subsume (LCS) of two words and their length are determined;
6. The similarity score between words using Eqs (16) and (17) is computed.

We use the following equations to calculate the semantic similarity between words [41, 42, 43, 44]:

$$IC\;\left(w\right)\;=1-\frac{Log\left(synset\left(w\right)+1\right)}{\text{log}\left(\text{max}\_\text{w}\right)}$$ (16)

$$Sim\left({\text{w}}_{1},{\text{w}}_{2}\right)\;=\{\begin{array}{l} \frac{2*\text{IC}\left(\text{LCS}\left({\text{w}}_{1},{\text{w}}_{2}\right)\right)}{\text{IC}\left({\text{w}}_{1}\right)+\text{IC}\left({\text{w}}_{2}\right)}\;\text{if}\;{\text{w}}_{1}\neq {\text{w}}_{2}\\ \;1\;\text{if}\;{\text{w}}_{1}={\text{w}}_{2} \end{array}$$ (17)

Where LCS stands for the least common subsume, max_w is the number of words in WordNet, Synset (*w*) is the number of synonyms of word w, and IC (*w*) is the information content of word w based on the lexical database WordNet.

Semantic similarity between sentences–We used semantic–vector approach [1, 45, 46] to measure the semantic similarity between sentences. The following tasks are performed to measure the semantic similarity between two sentences.

1. To create the semantic-vector.
  The semantic-vector is created using the word set and corresponding sentence. Each cell of the semantic-vector corresponds to a word in the word set, so the dimension equals the number of words in the word set.
2. To weight each cell of the semantic-vector.
  Each cell of the semantic-vector is weighted using the calculated semantic similarity between words from the word set and corresponding sentence. As an example:
  - If the word, *w*, from the word set appears in the sentence *S*_*1*_, the weight of the *w* in the semantic vector is set to 1. Otherwise, go to the next step;
  - If the sentence *S*_*1*_ does not contain the *w*, then compute the similarity score between the *w* and the words from sentence *S*_*1*_ using the *SSW* method.
  - If exist similarity values, then the weight of the *w* in the semantic-vector is set to the highest similarity value. Otherwise, go to the next step;
  - If there is no similarity value, then the weight of the *w* in the semantic-vector is set to 0.
3. The semantic-vector is created for each of the two sentences. The semantic similarity measure is computed based on the two semantic-vectors. The cosine similarity is used to calculate the semantic similarity between sentences:

$$Sim_{semantic}\left(S_{1}\;,S_{2}\right)=\frac{\sum_{j=1}^{m}\left(w_{1j}\times w_{2j}\right)}{\sqrt{\sum_{j=1}^{m}w_{1j}^{2}}\times \sqrt{\sum_{j=1}^{m}w_{2j}^{2}}}$$ (18)

Where S_1_ = (w_11_,w_12_,⋯,w_1m_) and S_2_ = (w_21_,w_22_,⋯,w_2m_) are the semantic vectors of sentences *S*_*1*_ and *S*_*2*_, respectively; *w*_*pj*_ is the weight of the *j*^*th*^ word in vector *S*_*p*_, *m* is the number of words.

Word order similarity between sentences–We use the syntactic–vector approach [47, 48] to measure the word-order similarity between sentences. The following tasks are performed to measure the word-order similarity between two sentences.

1. To create the syntactic-vector.
  The syntactic-vector is created using the word set and corresponding sentence. The dimension of current vector is equal to the number of words in the word set.
2. To weight each cell of the syntactic-vector.
  Unlike the semantic-vector, each cell of the syntactic-vector is weighted using a unique index. The unique index can be the index position of the words that appear in the corresponding sentence. However, the weight of each cell in syntactic-vector is determined by the following steps:
  - For each word, *w*, from the word set. If the *w* appears in the sentence *S*_*1*_ the cell in the syntactic-vector is set to the index position of the corresponding word in the sentence *S*_*1*_. Otherwise, go to the next step;
  - If the word *w* does not appear in the sentence *S*_*1*_, then compute the similarity score between the *w* and the words from sentence *S*_*1*_ using the *SSW* method.
  - If exist similarity values, then the value of the cell is set to the index position of the word from the sentence *S*_*1*_ with the highest similarity measure.
  - If there is not a similar value between the *w* and the words in the sentence *S*_*1*_, the weight of the cell in the syntactic-vector is set to 0.
3. For both sentences the syntactic-vector is created. Then, the syntactic similarity measure is computed based on the two syntactic-vectors. The following equation is used to calculate word-order similarity between sentences:

$$Sim_{\text{word order}}\left(S_{1},S_{2}\right)=1-\frac{\left|\left|O_{1}-O_{2}\right|\right|}{\left|\left|O_{1}+O_{2}\right|\right|}$$ (19)

Where O_1_ = (d_11_, d_12_,⋯, d_1m_) and O_2_ = (d_21_, d_22_,⋯, d_2m_) are the syntactic vectors of sentences *S*_*1*_ and *S*_*2*_, respectively; *d*_*pj*_ is the weight of the *j*^*th*^ cell in vector *O*_*p*_.

Sentence similarity measurement–The similarity measure between two sentences is calculated using a linear equation that combines the semantic and word-order similarity. The similarity measure is computed as follows:

$${\text{Sim}}_{\text{sentences}}\left({\text{S}}_{1},{\text{S}}_{2}\right)=\alpha \cdot {\text{sim}}_{\text{semantic}}\left({\text{S}}_{1},{\text{S}}_{2}\right)+\;\left(1-\alpha \right)\cdot {\text{sim}}_{\text{wordorder}}\left({\text{S}}_{1},{\text{S}}_{2}\right),\;0<\alpha <1$$ (20)

Where *alpha* is the weighting parameter, specifying the relative contributions to the overall similarity measure from the semantic and syntactic similarity measures. The larger the alpha, the heavier the weight for the semantic similarity. If alpha equals 0.5 the semantic and syntactic similarity measures are assumed to be equally important.

#### Sentences Relevance Detection Component (SRDC)

Let T_Original Text_ = {S_1_, S_2_⋯S_N_} represent all sentences from the original text, where *N* is the number of sentences. *S*_*s*_ denotes a summary sentence.

Let *Arr*_*Relations*_ *= {(S*_*1*_,*S*_*s*_,*Value*_*sim(S1*,*Ss)*_*)*,*(S*_*2*_,*S*_*s*_,*Value*_*sim(S2*,*Ss)*_ *)* ⋯*(S*_*M*_, *Ss*, *Value*_*sim(SM*,*Ss)*_ *)}* represent all the sentences from the original text that have relations with *S*_*s*_, where *M* is less than or equal to *N* and *Value*_*sim(SM*,*Ss)*_ indicates the similarity measure between two sentences *S*_*M*_ and *S*_*s*_.

Based on the previous section (*Intermediate-processing*), a summary sentence is related to any sentences of the original text, if the two sentences share at least a word. Hence, a set of sentences from the original text are found to have relations with a sentence of the summary text. Thus, it is important to determine which sentences from the source text have been used to create the summary sentence. In other words, we attempt to find a subset of the sentences *Arr*_*Relations*_ that are used to produce *Ss*. *Brr*_*Relevant sentences*_, *Brr*_*RS*_ represent a subset of the sentences *Arr*_*Relations*_. The steps to determine these sentences are as follows:

**Step 1**. It selects a relation from *Arr*_*Relations*_ with the greatest similarity score. Let *S*_*1*_ be a sentence of ArrRelations that has relation to *S*_*s*_ with the greatest similarity score, *Value*_*sim(S1*,*Ss)*_). Thus, this pair of sentences is taken to the next step.

**Step 2**. In the current step, all the common words between two sentences *S*_*1*_ and *S*_*s*_ are eliminated; then, the length of sentence *S*_*s*_ is checked. If it is equal to zero, it indicates that sentence *S*_*s*_ includes a phrase from one sentence in the original text and sentence *S*_*1*_ is used to create the sentence *S*_*s*_. In this case, sentence *S*_*1*_ is assigned to *Brr*_*RS*_ and then the cell (*S*_*1*_,*Ss*,*Value*_*sim(S1*,*Ss)*_) is removed from *Arr*_*Relations*_. Finally, the algorithm stops the current process. If the length of the sentence *S*_*s*_ is not equal to zero, the algorithm continues the process to the next step.

**Step 3**. Let *S*_*1*_^*'*^ represent sentence *S*_*1*_ with its remaining words and *S*_*s*_^*'*^ represent sentence *S*_*s*_ with its remaining words. Using the *SSW* method, the semantic similarity measure between the words of sentence *S*_*s*_^*'*^ and *S*_*1*_^*'*^ is calculated. If there is a similarity measure, the similar words would be removed. We then check the length of *S*_*s*_^*'*^. If it is equal to zero, this state shows that sentence *S*_*s*_ contains a phrase from one sentence in the original text, and that sentence *S*_*1*_ is used to create the sentence *S*_*s*_. Thus, sentence *S*_*1*_ is assigned to *Brr*_*RS*_ and then the cell (*S*_*1*_, *S*_*s*_, *Value*_*sim(S1*, *Ss)*_) is removed from *Arr*_*Relations*_. Finally, the algorithm stops the current process.

If the length of the sentence *S*_*s*_^*'*^ is not yet equal to zero, it shows that the sentence *S*_*s*_ contains a combination of phrases from two or more sentences in the original text. Thus, sentence *S*_*1*_ is assigned to *Brr*_*RS*_ and then the cell (*S*_*1*_, *S*_*s*_,*Value*_*sim(S1*,*Ss)*_) is removed from *Arr*_*Relations*_. Finally, the algorithm continues the process to the final step.

**Step 4**. In this step, to calculate sentence similarity and to find other sentences that are used to create sentence *S*_*s*_, *Arr*_*Relations*_^*'*^ with the remaining elements and sentence *S*_*s*_^*"*^ with the remaining words of *S*_*s*_^*'*^ are sent to the SSCC.

### Post–processing

The final step of ISSLK is to support the automatic assessment of summaries by identifying summarizing strategies. In fact, it aims, to answer the following questions:

1. What summarizing strategies have been used to create a summary sentence?
2. How can a topic sentence selection strategy be identified?
3. What are the methods used to identify a topic sentence selection strategy?

Table 3 summarizes the rules to identify each summarizing strategy and method. The overall processes for applying these rules to identify the summarizing strategies and methods are described as follows:

#### Identifying summarizing strategies used in summary writing

Deletion, sentence combination, copy-verbatim strategies–Given two texts, summary text and original text, Let *S*_*s*_ *= {W*_*1*_,*W*_*2*_⋯*W*_*K*_*}* be a sentence of the summary text and *Brr*_*RS*_ *= {(T*_*1*_, *S*_*s*_, *P*_*1*_*)*, *(T*_*2*_, *S*_*s*_, *P*_*2*_*)* ⋯*(T*_*N*_, *S*_*s*_, *P*_*M*_*)}* represent all the sentences from the original text that are used to produce sentence *S*_*s*_, where *k* is the number of words in *S*_*s*_, *M* is the number of phrases in the sentence *S*_*s*_, *T*_*N*_ is the *N*^*th*^ sentence from the original text and *(T*_*N*_, *S*_*s*_, *P*_*M*_*)* indicates that the *M*^*th*^ phrase of sentence *S*_*s*_ comes from the *N*^*th*^ sentence from the original text. The steps for identifying deletion, copy-pasting and sentence combination strategies are as follows:

**Step 1.** The algorithm checks the value of *N*. If it is equal to 1, then the algorithm attempts to find the deletion strategy and copy-verbatim strategy using step 2, otherwise, it attempts to identify the sentence combination strategy using step 3.

**Step 2.** Given two sentences, *T* and *Ss*, the algorithm computes the length of each sentence. Let *Len (T)* denote the length of sentence *T* and *Len (Ss)* denote the length of sentence *S*_*s*_. It also calculates the similarity measure between two sentences. Using *Len (T)*, *Len (Ss)* and *Sim (T, Ss)*, the following statements can be made:

$$State_{CP}=\left(\left(N=1)˄\left(Len(T)=Len(S_{s})\right)˄(Sim\;(T,S_{s})=1\right)\right)$$ (21)

$$State_{Del}=\left(\left(N=1\right)˄\left(Len(T)>Len(S_{s})\right)˄(Sim\;(T,S_{s})<1)\right)$$ (22)

Where *T* indicates a sentence of *Brr*_*RS*_ and *Sim (T*_,_ *S*_*s*_***)*** denotes the sentence similarity measure between *T* and *S*_*s*_.

The *State*_*CP*_ describes that the sentence *S*_*s*_ used the copy-verbatim strategy if one sentence is used to produce *S*_s,_ the length of two sentences is equal, and the similarity measure between two sentences is between 0 and 1 (but not 0).

The *State*_*Del*_ describes that sentence *S*_*s*_ used the deletion strategy and that if one sentence is used to produce *S*_*s*_, the length of sentence *S*_*s*_ is less than the length of sentence *T* and the similarity measure between two sentences is between 0 and 1 (but not 0 and 1). The algorithm also considers the two following rules to identify deletion strategy.

$$\forall \;W\;\in \;{\text{S}}_{s}|\;W_{O}\;\in \;T$$ (23)

Where, *W* is a word of *S*_*s*_ and *W*_*o*_ can be either a similar word or synonymous word.

$$\forall \;{\text{W}}_{1},{\text{W}}_{2}\;,{\text{W}}_{3}\in {\text{S}}_{\text{s}}\;:((\;{\text{W}}_{1}\;{\text{S}}_{\text{s}}\;{\text{W}}_{2}\wedge \;{\text{W}}_{2}\;{\text{S}}_{\text{s}}\;{\text{W}}_{3})\in {\text{S}}_{\text{s}})\Rightarrow \;(({\text{W}}_{1}\;{\text{S}}_{\text{o}}\;{\text{W}}_{2}\;\wedge \;{\text{W}}_{2}\;{\text{S}}_{\text{o}}\;{\text{W}}_{3})\in \text{T})$$ (24)

Where,

*W*_*1*_ *S*_*s*_ *W*_*2*_: *W*_*2*_ appears after *W*_*1*_ in sentence *S*_*s*_.

*W*_*2*_ *S*_*s*_ *W*_3:_ *W*_*3*_ appears after *W*_*2*_ in sentence *S*_*s*_.

*W*_*1*_ *S*_*o*_ *W*_*2*_: *W*_*2*_ appears after *W*_*1*_ in sentence *T*.

*W*_*2*_ *S*_*o*_ *W*_*3*_: *W*_*3*_ appears after *W*_*2*_ in sentence *T*.

**Step 3.** If the value of *N* is greater than 1, it indicates that more than one sentence from the original text is used to produce the sentence *S_s_*. Hence, the *S_s_* used the sentence combination strategy if the value of *N* was greater than 1 and the average of the semantic similarity measure is between 0 and 1 (but not 0). The corresponding statement is provided below:

$$State_{sentence\;combination}=\left((N>1)˄\left(\;\frac{\sum_{J=1}^{i}\mathrm{Sim}\left(s_{r}{}_{j},\;S_{s}\right)}{\mathrm{TN}}<1\right)\right)$$ (25)

Since the summary sentence *S*_*s*_ contains a combination of phrases from two or more sentences in the original text, each phrase of sentence *S*_*s*_ can be analyzed to identify other summarizing strategies, such as deletion, copy-pasting, topic sentence selection and paraphrasing.

Paraphrase strategy–Given two sentences, let *S*_*summary*_ *= {W*_*1*_, *W*_*2*_, ⋯*W*_*N*_*}* be a sentence of a summary text, where *N* is the number of words in the sentence *S*_*summary*_, *S*_*RS*_ *= {W*_*1*_, *W*_*2*_, ⋯*W*_*M*_*}* be a sentence of *Brr*_*RS*_ that is used to create the sentence *S*_*summary*_, where *M* is the number of words in sentence *S*_*RS*_. *A*_*Root*_ *= {W*_*R1*_, *W*_*R2*_,⋯*W*_*RN*_*}* includes the root of each word of sentence *S*_*summary*_, where *W*_*Rj*_ is the root of *j*^*th*^ word in sentence *S*_*summary*_.

*B*_*Synonym*_ *= {W*_*1*_, *W*_*2*_,⋯*W*_*K*_*}*includes the synonym of each word of the sentence *S*_*summary*_. In the first step, the algorithm by a loop for each word of sentence *S*_*RS*_ obtains the root and the synonyms using WordNet, then assign them to *A*_*Root*_ and *B*_*Synonym*_, respectively.

In the second step, the algorithm by a loop for each word of sentence *S*_*summary*_ determines the root of the word using the WordNet. Let *RW* be the root of the word. It checks if the *RW* was in *A*_*Root*_, and then continues the loop by the next word, otherwise, it searches for *RW* in *B*_*Synonym*_, then, if the search result is true, it indicates that the sentence *S*_*summary*_ used the paraphrase strategy, and the current loop will then stop.

Topic sentence selection strategy: cue, title, keyword, location methods–Given two sentences, let

1. S_summary_ be a sentence of summary text, *S*_*RS*_ be a sentence of *Arr*_*Relevant sentences*_ that is used to produce the sentence *S*_*summary*_;
2. L_cue word_ = {CW_1_,CW_2_, ⋯ CW_N_} denote a list of cue words;
3. L_key word_ = {KW_1_, KW_2_, ⋯KW_k_} denote a list of keywords;
4. L_title word_ = {TW_1_,TW_2_, ⋯TW_M_} denote a list of title words;
5. L_sentence location_ = {(S_1_,L_B_,L_E_),(S_2_,L_B_,L_E_), ⋯S_j_, L_B_,L_E_)} denote the location of the sentences in the source text, where *L*_*B*_ indicates the first sentence of a paragraph, *L*_*E*_ indicates the last sentence of a paragraph, and *(S*_*j*_, *L*_*B*_, *L*_*E*_*)* indicates that the *j*^*th*^ sentence, *S*, from source text is the first or the last sentence of a paragraph. Usually, those sentences are at the beginning and end of a document, the first and last sentences of paragraphs and also immediately below section headings. The steps for identifying the topic sentence selection (TSS) strategy using the four methods, cue, title, location and keyword are identified as follows:

Title method–In the first step, it checks the sentence *S*_*RS*_ for identifying the title method. Thus, if a word of *L*_*title word*_ is in sentence *S*_*RS*_, it indicates that the sentence *S*_*summary*_ used the title method; otherwise it did not use this method.

Key word method–In the second step, it checks the sentence *S*_*RS*_ for identifying the keyword method. Thus, if a word of the *L*_*key word*_ is in the sentence *S*_*RS*_, it indicates that the sentence *S*_*summary*_ used the keyword method; otherwise it did not use this method.

Location method—In the third step, it checks the sentence *S*_*RS*_ for identifying the location method. Thus, if the sentence *S*_*RS*_ is in L_sentence location_, it indicates that the sentence *S*_*summary*_ used the location method, otherwise it did not use this method.

Cue method–In the fourth step, it checks sentence *S*_*RS*_ to identify the cue method. Thus, if a word of *L*_*cue word*_ is in sentence *S*_*RS*_, it indicates that the summary sentence *S*_*summary*_ used the cue method; otherwise it did not use this method.

Finally, the sentence *S*_*summary*_ used topic sentence selection if it used at least one of these methods–keyword, cue, title and location.

## Experimental Evaluations

To evaluate the ISSLK algorithm, we carried out two experiments. In the first experiment, we measured the performance of the algorithm against human judgment to identify the summarizing strategies. In second experiment, we compare the performance of the algorithm with the existing method. To do this, we now explain our experiments on the single-document summarization datasets provided by Document Understanding Conference (DUC) (http://duc.nist.gov).

### Data set

In this section, we describe the data that used throughout our experiments. For assessment of the performance of the proposed method we used the document datasets DUC 2002 and corresponding 100-word summaries generated for each of documents. DUC 2002 contains 567 documents-summary pairs from Document Understanding Conference. It is worth mentioning that each document of DUC 2002 is denoted by original text or source text and the corresponding summary is denoted by candidate summary. We also used a set of students’ summaries. In our experiments, the documents and corresponding summaries were randomly divided into two separate dataset. Table 4 gives a brief description of the datasets.

**Table 4:** Description of dataset.

DUC 2002	
Number of cluster	59
Number of documents in each cluster	~ 10
Number of documents	567
Data source	TREC
Summary length	100 words

### Evaluation Metric

To evaluate the performance of the ISSLK, an evaluation metric is required. Various evaluation metrics are widely used in different natural language processing applications. In our experiment, the evaluation is performed using precision, recall and F-measure.

#### Precision, Recall and F–score

Precision, recall and F-score are the prevalent measures for evaluating a system [49]. Precision is the fraction of selected items that are correct and recall is the fraction of correct items that are selected. In this work, the summarizing strategies identified by a human refer to a set of ideal items, and the strategies identified by an algorithm refer to a set of system items. Precision is used to assess the fraction of the system items that the algorithm correctly identified and recall is used to assess the fraction of the ideal items that the algorithm identified. The precision is computed using Eq 26. It is the division of identified summarizing strategies by ISSLK and human expert over the number of summarizing strategies identified by Algorithm only. The recall is computed using Eq 27. It is the division of identified summarizing strategies by ISSLK and human expert intersection over the number of summarizing strategies identified by human expert.

$$\text{Pericision}=\frac{A}{A+B}$$ (26)

$$\text{Recall}=\frac{A}{A+C}$$ (27)

Where,

*A* = The number of summarizing strategies identified by Algorithm and Human expert.

*B* = The number of summarizing strategies identified by Algorithm only.

*C* = The number of summarizing strategies identified by Human expert only.

There is an anti–correlation between precision and recall (Manning et al., 2008). It means, the recall drops when the precision drops and vice versa. To take into consideration the two metrics together, a single measure, called F-score, is used. F-score is a statistical measure that merges both precision and recall. It is calculated as follows:

$$\text{F}-\text{measure}=\frac{1}{\alpha \times \frac{1}{P}+\left(1-\alpha \right)\frac{1}{R}}=\frac{\left(\beta^{2}+1\right)P\times R}{\beta^{2}\times P+R},\;\beta^{2}=\frac{1-\alpha }{\alpha }\;,\;\alpha \in \left[0\;,\;1\right],\;\beta^{2}\in \left[0\;,\infty \right]$$ (28)

If a large value assigns to the beta, it indicates that precision has more priority. If a small value assigns to the beta it indicates that recall has more priority. If beta is equal to 1 the precision and recall are assumed to have equally priority in computing F-score. F-score for beta equals 1 is computed as follows:

$$\text{F}-\text{measure}=\frac{2\times P\times R}{P+R}$$ (29)

Where *P* is precision and *R* is recall.

### Experiment 1—Evaluation of the algorithm with the human judgment

#### Procedure

Method H_0_ –Summary Text—Source text. One method that can be used to identify the strategies employed by the summarizer is as follows. The first split the summary text into a number of sentences. The second, for each summary sentence determine all relevant sentences from the source text that are associated to produce the current summary sentence. Finally, ccompare the current summary sentence and the all relevant sentences from the source text to identify the strategies used to produce the current summary sentence.

To evaluate the algorithm, we need a gold standard data, which is a set of all correct results. Based on this dataset, also known as judgment data, we can decide whether the output of the algorithm is correct or not. For this purpose, two experts: a) An English teacher with good reading skills and understanding ability in the English language as well as experience in teaching summary writing; b) A lecturer with experience in using the skills in their teaching method, were asked to identify the summarizing strategies used by summarizer in each summary sentence. Once the subjects completed the task using method H_0_, we compared the results, the summarizing strategies identified by the ISSLK with those identified by subjects. Table 5 shows summarizing strategies identified ISSLK and Human expert as an example.

**Table 5:** Summarizing strategies identified by RDSSIA and Human expert.

	Summarizing Strategies / Methods Identified	
Summary sentences	Human expert	ISSLK
“My father dived and swam as hard as he could to the spot where i had gone under.”	Deletion; Key word; T.S.S	Deletion; Sentence combination; Key word; T.S.S
“I gasped for air in desperation; the salty water filled my throat and nostrils.”	Deletion; Title word; T.S.S	Deletion; Title word; T.S.S
“The currents kept pushing the boat further and further away.”	Deletion; Key word; Location; Cue; T.S.S	Deletion; Key word; Location; T.S.S
“I was determined not to lose it.”	Location; T.S.S; Copy- verbatim	Copy-verbatim
“I felt myself sinking to the bottom and my father save me.”	Deletion; Sentence combination; Key word; T.S.S; Invention	Deletion; Sentence combination; Paraphrase; Key word; Location; T.S.S
“I was determined not to go lose it and I stretched my arm as far as it could go and tried to grab the boat.”	Deletion; Sentence combination; Key word; Location; Title word; T.S.S; Copy-verbatim	Deletion; Sentence combination; Key word; Title word; T.S.S

We used Cohen's Kappa [50, 51] as a measure of agreement between the two raters. The Kappa coefficient for measuring the inter-raters agreement was 0.61. This value indicated that our assessors had good agreement [52] for grading each student summary.

#### Parameter setting

The proposed algorithm requires parameter to be determined before use: a weighting parameter (*alpha*) (refer to Eq 20) for weighting the significance between semantic information and syntactic information. The parameter in the current experiment was found using training data. We ran our proposed algorithm, ISSLK, on the training dataset. We evaluate ISSLK for each *alpha* between 0.1 to 0.9 with a step of 0.1. Table 6 presents our experimental results obtained by using various the alpha values. We evaluate the results in terms of precision, recall and F-measure. By analyzing the results, we find that the best performance is achieved by an alpha value 0.7. This alpha produced the scores for three metrics as follows: 0.8126 (precision), 0.6818 (recall), 0.7415 (F-measure). The best values of Table 6 have been marked in boldface. As a result, using the current data set, we obtain the best result when we use 0.7 as the alpha value. Therefore, we can recommend this the alpha values for use on the testing data.

**Table 6:** Comparison between human and RDSSIA against various α values.

Weighting (α)	Precision	Recall	F-score
0.1	0.6229	0.5381	0.5774
0.2	0.6312	0.5340	0.5785
0.3	0.6404	0.5760	0.6065
0.4	0.6525	0.5934	0.6215
0.5	0.6867	0.5800	0.6289
0.6	0.7216	0.6922	0.7066
0.7	0.8126	0.6818	0.7415
0.8	0.7432	0.7094	0.7259
0.9	0.7559	0.6354	0.6904

#### Performance analysis

To confirm the aforementioned results, we validate our proposed algorithm, ISSLK. To do this, we measure the performance of the algorithm against human judgment to identify the summarizing strategies using unused data set, testing data. We apply ISSLK to the testing data set only with the alpha value 0.7. To compute the precision, recall and F-measure, we determine the values of *A*, *B* and *C* by analysing the number of summarizing strategies identified by the algorithm and human (*A*), the number of summarizing strategies identified by algorithm only (*B*), and the number of summarizing strategies identified by human only (*C*). Then, the equations of precision, recall and F-measure are applied to obtain the values for each summary.

## Results and Discussion

According to the results presented in Table 7, the algorithm obtained an average of 77% precision, 66% recall and 70% F-score for summaries. It did not attain a high percentage for the precision, recall and F-score in comparison to human judgment due to various reasons, such as:

1. The algorithm failed to identify some of the summarizing strategies identified by the expert. These strategies are generalization and invention. It has affected the result of the algorithm and is the reason why we did not achieve the high percentage for precision and finally F-score. However, this limitation is understandable because the algorithm was designed to identify the summarizing strategies and methods–paraphrase, topic sentence selection, sentence combination, copy–verbatim, key–words method, title method, location method and cue method–and is not able to identify strategies such as invention and generalization.
2. Another reason is that when the algorithm and human want to identify the topic sentence selection strategy using the cue method. The cue method used cue words, such as “in conclusion” and “as result”, to display the important sentence in a text. These cue words rely on the content of the text. Thus, it is difficult to derive the list of cue words, since different types of text may generate a different list of cue words. Hence, there is no standard list of cue words; the lack of this standard list affects the results of the algorithm.
3. The algorithm used WordNet as the main semantic knowledge base for the calculation of semantic similarity between words. The comprehensiveness of WordNet is determined by the proportion of words in the text that are covered by its knowledge base. However, the main criticism of WordNet concerns its limited word coverage to calculate semantic similarity between words. Obviously, this disadvantage has a negative effect on the performance of our proposed algorithm.
4. The algorithm is not able to distinguish between an active sentence and a passive sentence. Given a summary sentence (A: ‘*Father likes his child*.*’*) and two original sentences (B: ‘*child likes his father*.*’; C*: ‘*child is liked by his father*.*’*), although the similarity measure between sentences (*A* and *B*) and (*A* and *C*) is same, but as we can see the meaning of sentence *A* is more similar to the sentence *C*. hence, it is important to know what passive and active sentences are before comparisons can be drawn.

**Table 7:** Precision, Recall and F-score, (Due to space limitations of this paper, a sample results are shown).

Summary	A	B	C	Precision	Recall	F-score
1	4	3	2	0.57	0.67	0.62
2	9	0	3	1.00	0.69	0.82
3	8	0	7	1.00	0.47	0.64
.	.	.	.	.	.	.
.	.	.	.	0.77	0.66	0.70

### Experiment 2—Comparison with related methods

In this section, the performance of our algorithm is compared with other well-known or recently proposed methods. In particular, to evaluate our methods on data set, we select the following methods: SSDA [15] and MSAS [14]. The evaluation metrics values are presented in Tables 8 and 9. In Table 9 ‘‘- - -” means the proposed method could not identify the corresponding summarizing strategies. The above mentioned approaches use different data sources in their experiments. This makes a direct comparison between evaluation results of the different approaches impossible. In addition, they used different evaluation measures. Therefore, we re-examined the mentioned approaches upon the same dataset.

**Table 8:** Performance comparison between ISSLK and other methods.

System	Precision	Recall	F-score
ISSLK	0.86	0.81	0.83
MSAS	0.81	0.78	0.79
SSDA	0.76	0.68	0.72

**Table 9:** Performance comparison between ISSLK and other methods.

Systems	Metrics	Copy-verbatim	Deletion	Paraphrasing	Sentence Combination	T.S.S
ISSLK	Precision	0.89	0.91	0.90	0.94	0.87
	Recall	0.82	0.87	0.84	0.89	0.79
	F-measure	0.85	0.89	0.87	0.91	0.83
MSAS	Precision	0.84	- - -	0.83	- - -	- - -
	Recall	0.77	- - -	0.78	- - -	- - -
	F-measure	0.80	- - -	0.80	- - -	- - -
SSDA	Precision	0.79	0.6	- - -	0.77	- - -
	Recall	0.74	0.57	- - -	0.73	- - -
	F-measure	0.76	0.58	- - -	0.75	- - -

#### Detailed comparison

With comparison to the precision and F-score values for other methods, our proposed method achieved significant improvement. Table 10 shows the improvement of ISSLK for all two metrics. It is clear that ISSLK obtains the high F-measure values and outperforms all the other methods. We use the relative improvement, Eq 30, for comparison. In Table 10 ‘‘+” means the proposed method improves the related methods. Table 10 presents among other methods the MSAS shows the best results compared to SSDA. Compared with the method MSAS, our method improves the performance by (6.1728) %, and (4.9746) % in terms precision and F-score metrics, respectively.

$$\text{Improvement}=(\frac{\text{Our method}-\text{Other method}}{\text{Other method}})\;\times \;100$$ (30)

**Table 10:** Performance evaluation compared between the ISSLK and other methods.

System	Precision	F-score
MSAS	+ 6.1728	+ 4.9746
SSDA	+ 13.1578	+ 16.2269

## Conclusion

Summarizing strategies are the core of the cognitive processes involved in the summarization activity. In this paper, we propose an algorithm based on the linguistic measure to identify the summarizing strategies used by summarizer in summary writing. The algorithm employs three similarity metrics to calculate similarity measure between two sentences: *a)* semantic similarity between sentences; *b)* word-order similarity between sentences; and *c)* semantic similarity between words. The main feature of the proposed algorithm is its ability to capture the meaning in comparison between a source text sentence and a summary text sentence, when two sentences have same surface text or different words have been used in the sentences. This algorithm is also able to identify summarizing strategies at both the semantic and syntactic levels. The algorithm is able to identify summarizing strategies and methods such as deletion, sentence combination, paraphrase, copy-verbatim, topic sentence selection, cue method, title method, keyword method and location method.

The evaluation of ISSLK is conducted over DUC dataset. The proposed algorithm is very easy to follow and requires minimal text processing cost. Initially, parameter of ISSLK is optimized over the training dataset. Later the actual summarizing strategies identification evaluation is done over test dataset. The first experiment was conducted to evaluate the performance of the algorithm using the comparison between the algorithm and human judgments. The result demonstrates that the algorithm obtained an average of 77% precision, 66% recall, 70% F-score. ISSLK is compared with the current systems which are well-known existing systems that are proposed to identify summarizing strategies. The experimental results display that the performance of the proposed algorithm is very competitive when compared with other systems. The results also displayed that ISSLK improved the performance of the existing system. We observed that ISSLK is able to obtain an average of 86% precision, 81% recall, 83% F-score.

This paper presents the following suggestions for future work. Firstly, the algorithm failed to identify some of the strategies, such as generalization and invention. To improve the performance of the algorithm in identifying summarizing strategies, it needs to work on algorithm to identify the strategies, such as generalization and invention. Obviously, this can improve the precision, recall, F-measure, and, finally, the accuracy of the algorithm. Finally, we are confident that the idea of incorporating semantic and syntactic information can be further explored by using a combination of more complex techniques and modules for text analysis. This is because once a passive or active sentence has been used in writing, it is important to know what passive and active sentences are before comparisons can be drawn. Finally, our method used WordNet as the main semantic knowledge base for the calculation of semantic similarity between words. The comprehensiveness of WordNet is determined by the proportion of words in the text that are covered by its knowledge base. However, the main criticism of WordNet concerns its limited word coverage to calculate semantic similarity between words. Obviously, this disadvantage has a negative effect on the performance of our proposed method. One solution is that, in addition to WordNet, other knowledge resources must be used.

In addition future works, we aim to examine other method to compute semantic similarity between words. It can be useful for increasing the overall performance of the proposed method.

## Supporting Information

S1 Dataset Used to evaluate the proposed algorithm. (XML)
